# Tissue‐Engineered Neural Network Graft Relays Excitatory Signal in the Completely Transected Canine Spinal Cord

**DOI:** 10.1002/advs.201901240

**Published:** 2019-09-19

**Authors:** Bi‐Qin Lai, Ming‐Tian Che, Bo Feng, Yu‐Rong Bai, Ge Li, Yuan‐Huan Ma, Lai‐Jian Wang, Meng‐Yao Huang, Ya‐Qiong Wang, Bin Jiang, Ying Ding, Xiang Zeng, Yuan‐Shan Zeng

**Affiliations:** ^1^ Key Laboratory for Stem Cells and Tissue Engineering (Sun Yat‐sen University) Ministry of Education Guangzhou 510080 China; ^2^ Department of Histology and Embryology Zhongshan School of Medicine Sun Yat‐sen University Guangzhou 510080 China; ^3^ Institute of Spinal Cord Injury Sun Yat‐sen University Guangzhou 510120 China; ^4^ Co‐innovation Center of Neuroregeneration Nantong University Nantong 226001 China; ^5^ Guangdong Provincial Key Laboratory of Brain Function and Disease Zhongshan School of Medicine Sun Yat‐sen University Guangzhou 510080 China; ^6^ Department of Electron Microscope Zhongshan School of Medicine Sun Yat‐sen University Guangzhou 510080 China

**Keywords:** canine, neural network tissue, neuronal relay, spinal cord injury, TrkC

## Abstract

Tissue engineering produces constructs with defined functions for the targeted treatment of damaged tissue. A complete spinal cord injury (SCI) model is generated in canines to test whether in vitro constructed neural network (NN) tissues can relay the excitatory signal across the lesion gap to the caudal spinal cord. Established protocols are used to construct neural stem cell (NSC)‐derived NN tissue characterized by a predominantly neuronal population with robust trans‐synaptic activities and myelination. The NN tissue is implanted into the gap immediately following complete transection SCI of canines at the T10 spinal cord segment. The data show significant motor recovery of paralyzed pelvic limbs, as evaluated by Olby scoring and cortical motor evoked potential (CMEP) detection. The NN tissue survives in the lesion area with neuronal phenotype maintenance, improves descending and ascending nerve fiber regeneration, and synaptic integration with host neural circuits that allow it to serve as a neuronal relay to transmit excitatory electrical signal across the injured area to the caudal spinal cord. These results suggest that tissue‐engineered NN grafts can relay the excitatory signal in the completely transected canine spinal cord, providing a promising strategy for SCI treatment in large animals, including humans.

## Graphical abstract

In vitro constructed engineered neural network tissue, characterized by a neuronal population with robust trans‐synaptic activities, can survive within the completely transected canine spinal cord, integrate with host neural circuits, and serve as a neuronal relay to transmit excitatory electrical signals across the injured area to the caudal spinal cord, leading to significant motor recovery within the paralyzed pelvic limbs.

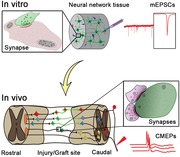

## 1 Introduction

Spinal cord injury (SCI) causes irreversible tissue loss, including neurons in the gray matter and nerve tracts and oligodendrocytes in the white matter.^1^ Spontaneous regeneration after SCI is limited, and there are no satisfactory interventions to enhance endogenous regeneration to replenish lost tissue, resulting in functional deficits below the level of injury.^2^, ^3^ Reconstruction of neural tissue in the injured area using exogenous neural grafts may supplement the injury gap with an appropriate neural population to facilitate motor, sensory, and autonomic functional recovery.^4^, ^5^ The rapid development of stem cell therapy and tissue engineering technology offers a promising therapeutic strategy for SCI.^6^ For example, neurons and glia derived from grafted human neural stem cells (NSCs) were able to replace lost neurons and glia excised by hemisection surgery, and they functioned as interneurons to reconnect severed neural circuits in a rodent SCI model.^7^

A prodifferentiation regimen is required to increase neuronal yield from NSCs, either endogenous or exogenous, because they are prone to differentiate into astrocytes in the post‐SCI milieu.^8^ When NSCs were delivered in a fibrin–thrombin matrix containing trophic factors including brain‐derived neurotrophic factor (BDNF), neurotrophin‐3 (NT‐3), platelet‐derived growth factor (PDGF‐AA), insulin‐like growth factor 1 (IGF‐1), epidermal growth factor (EGF), basic fibroblast growth factor (bFGF), acidic fibroblast growth factor (aFGF), glial cell line‐derived neurotrophic factor (GDNF), hepatocyte growth factor (HGF), and calpain inhibitor, they mainly differentiated into neurons in the spinal cord.^9^, ^10^ However, concerns were raised regarding the risk of long‐distance migration and ectopic neurogenesis by donor NSCs or neural progenitor cells that were transplanted immediately after SCI modeling in a trophic factor‐enriched microenvironment.^11^ Thus, an alternative approach would be to augment in situ neuronal differentiation at the injury site through application of functionally defined single reagents such as the histone deacetylase inhibitor valproic acid (VPA),^12^ the EGF receptor signaling antagonist cetuximab,^13^ the microtubule‐stabilizing agent paclitaxel,^14^ and others. To drive neuronal differentiation of NSCs with minimal adverse effects on host homeostasis, the doses of these adjuvant compounds should be fine‐tuned and their release should be spatiotemporally controlled, which is a substantial engineering challenge.

Our team has established a tissue engineering methodology to construct neural network (NN) tissue in vitro using biocompatible bioscaffolds, NSCs, and lentiviral‐based trophic factor delivery.^15^ Our previous results showed that NSCs expressing a genetically modified version of NT‐3 receptor TrkC can be induced to differentiate into neurons with mature neuronal properties including firing action potentials and trans‐synaptic electrochemical activities when they were cocultured with Schwann cells (SCs) with genetically modified NT‐3 in a 3D gelatin sponge scaffold. Together with deposited extracellular matrix (ECM), SCs and NSC‐derived neurons formed a homeostatic system allowing for intercellular interactions between cells and the ECM. This tissue engineering construct functions as NN tissue.^16^ Previous studies showed that prebuilt NN tissue survived the hostile post‐SCI microenvironment for up to 2 months and acted as a relay to repair neural circuits in a rat‐transected SCI model without ectopic colony formation.^17^, ^18^ Results from rat SCI models support our hypothesis that transplantation of a tissue engineering NN tissue is a promising therapeutic approach to repair SCI.

However, given significant interspecies differences in terms of anatomy, pathology and pathophysiology, findings derived from rodent models may not generalize to humans.^19^, ^20^ Therefore, large animal models should be used before clinical studies to bridge the gap between rodent and human studies. The canine model has several innate advantages for studying therapeutic SCI interventions.^21^, ^22^ First, canines with SCI underwent similar pathological processes and repair mechanisms as observed in patients.^23^ Compared with other large animal SCI models, postoperative care is easier to administer in canines with SCIs. Moreover, domesticated canines are more compliant with multimodal behavioral tests than other large animals, allowing for more reliable evaluation of therapeutic effects.^21^, ^24^ We recently established a complete SCI canine model and tested the therapeutic effects of canine bone marrow mesenchymal stem cell (MSC)‐derived NN tissue on motor function recovery.^16^ Although MSC‐derived NN tissues exhibit potent therapeutic efficacy and translational value, the mixed population of neuron‐like cells and other MSC‐derived cell populations made analysis of functional recovery mechanisms difficult. How exogenous neurons participate in repairing damaged neural circuits in large animals with considerable spinal cord tissue loss remains to be determined. Therefore, we adopted an established complete SCI canine model to assess the effects of NSC‐derived NN tissue on SCIs with large tissue deficits. Our aim was to evaluate whether and how tissue engineered NSC‐derived NN tissues could integrate with host neural circuits and serve as neuronal relays to help cortex excitatory signal cross the lesion gap to the caudal spinal cord.

## 2 Results

### 2.1 Canine NSCs and SCs Culture and Gene Modification

NSCs were isolated from the hippocampus, and nestin‐positive neurospheres were transfected with a lentivirus carrying a TrkC coding sequence (pLent‐EF1a‐TrkC‐Flag‐CMV‐GFP‐P2A‐Puro). After transfection, nearly all cells within neurospheres were GFP positive, with most expressing the NSC marker nestin (Figure S1A, Supporting Information). SCs were bipolar in shape after two passages. S100 immunofluorescence staining showed that ≈90% of the bipolar cells were SCs (Figure S1B, Supporting Information). SCs were genetically modified with an NT‐3 lentivirus (pLent‐EF1a‐NT‐3‐Flag‐CMV‐P2A‐Puro). After 14 d of coculture in collagen sponge (CS) scaffolds, > 90% of NSCs maintained TrkC and GFP expression (T‐NSCs, Figure S1C, Supporting Information). Most SCs (GFP negative) maintained NT‐3 expression (N‐SCs, Figure S1D, Supporting Information). The experiment groups were as follows: NSCs, NSCs+SCs, T‐NSCs+SCs, NSCs+N‐SCs, and T‐NSCs+N‐SCs.

### 2.2 Phenotypic and Functional Identification of NSC‐Derived NN Tissue In Vitro

**Figure 1:**
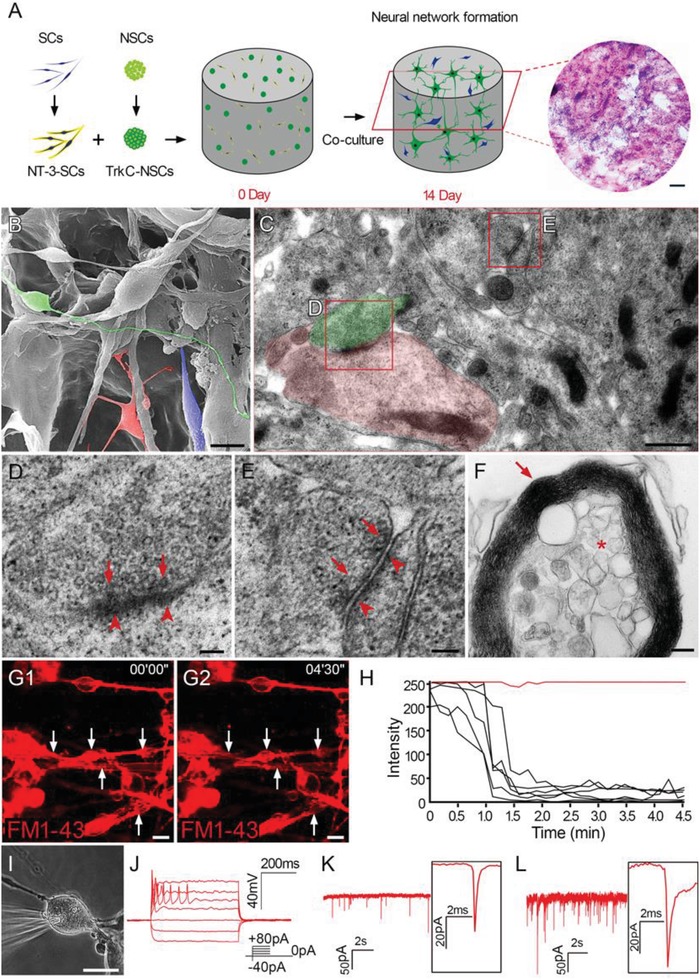
Construction of canine NSC‐derived NN tissue. A) Schematic diagram illustrating construction of canine NSC‐derived NN tissue in a 3D CS scaffold. NSCs with genetically modified TrkC (TrkC‐NSCs) were cocultured with SCs with genetically modified NT‐3 (NT‐3‐SCs) in the 3D CS scaffold for 14 d. The H&E staining revealed the cross section of the 3D collagen sponge containing cells. B) SEM showing neuron‐like cells with long processes (green), oligodendrocyte‐like cells with multiple short processes wrapping other processes (red), and SC‐like cells (blue) in NSC‐derived NN tissue. C–E) TEM images showing features of synapses (presynaptic element, green in (C) and postsynaptic element, red in (C)) between processes of two neurons. These were characterized by the presence of vesicular profiles (arrows in (D) and (E)) in one process and a layer of electron‐dense structures (arrowheads in (D) and (E)) in the other. F) Multilamellar myelin sheaths (arrows) wrapping an axonal profile (asterisk) in the NN tissue. G1,G2) Neurons unloaded prelabeled FM1‐43 dye (red, white arrows) following membrane depolarization triggered by high [K**^+^**] stimulation, as shown by H) the steep drop of fluorescence intensity after stimulation. I) Whole cell patch‐clamp recording showed action potentials of neurons following J) 21 d of culture. K) High‐frequency miniature excitatory postsynaptic currents (mEPSCs) and L) miniature inhibitory postsynaptic currents (mIPSCs) were detected. Scale bars = 500 µm in (A); 10 µm in (B); 0.5 µm in (C); 100 nm in (D) and (E); 200 nm in (F); 20 µm in (G1), (G2), and (I).

To construct NSC‐derived NN tissues, T‐NSCs and N‐SCs were cocultured in a 3D CS scaffold for 14 d (the T‐NSCs+N‐SCs group, **Figure** **1**A). Immunofluorescence staining of slices from the T‐NSCs+N‐SCs group showed that NSCs differentiated into neurons expressing microtubule‐associated protein 2 (Map2, a neuronal marker) and postsynaptic density protein 95 (PSD95, a postsynaptic marker, Figure S1E, Supporting Information). These neurons also showed immunoreactivity for choline acetyl transferase (ChAT, a cholinergic marker, Figure S1F,M, Supporting Information), glutamate decarboxylase 67 (GAD67, a GABAergic marker, Figure S1G,M, Supporting Information), and Ca^2+^/calmodulin‐dependent protein kinase 2α (CaMK2α, an excitatory marker, Figure S1H,M, Supporting Information). SCs within the NN tissue were GFP negative and expressed myelin basic protein (MBP, a myelin marker, Figure S1I, Supporting Information) or glial fibrillary acidic protein (GFAP, an astrocyte marker, Figure S1J, Supporting Information).

Western blot analysis showed that protein levels of neurofilament 200 (NF, a neuron marker), PSD95, synaptophysin (SYP, a presynaptic marker), ChAT, and GAD67 were highest in the T‐NSCs+N‐SCs group (Figure S1N,O, Supporting Information), suggesting that T‐NSCs had the greatest levels of neurogenesis, synapse formation, and neurotransmitter synthesis when cocultured with N‐SCs in vitro. MBP expression levels indicated that the T‐NSCs+N‐SCs group had the highest potential for myelination in vitro (Figure S1N,O, Supporting Information).

Scanning electron microscope (SEM) analysis showed extensive contacts between long processes of SC‐like, oligodendrocyte‐like, and neuron‐like cells (Figure 1B). Synaptic features, such as synaptic vesicles at axonal terminals, and PSD formation were detected in neurons within the NN tissue using transmission electron microscope (TEM, Figure 1C–E). High magnification of the boxed areas revealed the details of the relatively mature synapse (Figure 1D), and relatively immature symmetrical synapses (Figure 1E). The results suggest dynamic changes in the formation synapses in NN tissue. Furthermore, multiple lamellar structures appeared to form myelin sheaths around axons in the NN tissue (Figure 1F).

When a high [K^+^] solution containing *N*‐3‐triethylammonmpropyl‐4‐4‐(dibutylamino) styryl (FM1‐43) dye was added to the culture dish containing the T‐NSCs+N‐SCs scaffold, the cells endocytosed the FM1‐43 dye, rendering the membrane red (Figure 1G1) when visualized using a fluorescence microscope. When these cells were restimulated with a high [K^+^] solution without FM1‐43 dye, fluorescence intensity rapidly decreased (Figure 1G2,H), indicating that the cells had the ability to quickly exocytose FM1‐43 dye in a manner similar to synaptic vesicle release.^25^ Based on these findings, whole‐cell patch clamp was used to evaluate the electrophysiological properties of NSC‐derived neuron‐like cells (Figure 1I,J). Evoked action potentials were recorded in 8/10 neuron‐like cells in the T‐NSCs+N‐SCs group. Postsynaptic currents including miniature excitatory postsynaptic currents (mEPSCs) and miniature inhibitory postsynaptic currents (mIPSCs) were detected (Figure 1K,L), suggesting trans‐synaptic communication.

These findings suggest that canine NSCs expressing genetically modified TrkC differentiated into neurons with neuronal phenotypes, synaptic structure, and electrophysiological function when cocultured with SCs with genetically modified NT‐3 in the 3D CS scaffold for up to 14 d. The scaffold provided a matrix for cell adherence and growth, and an anchoring surface for ECM deposition (Figure S1K,L, Supporting Information). This allowed differentiating cells, their surrounding ECM, and the CS to develop into NN tissue after 14 d of culture.

### 2.3 Transplantation of NSC‐Derived NN Tissue Improved Hindlimb Motor Function

**Figure 2:**
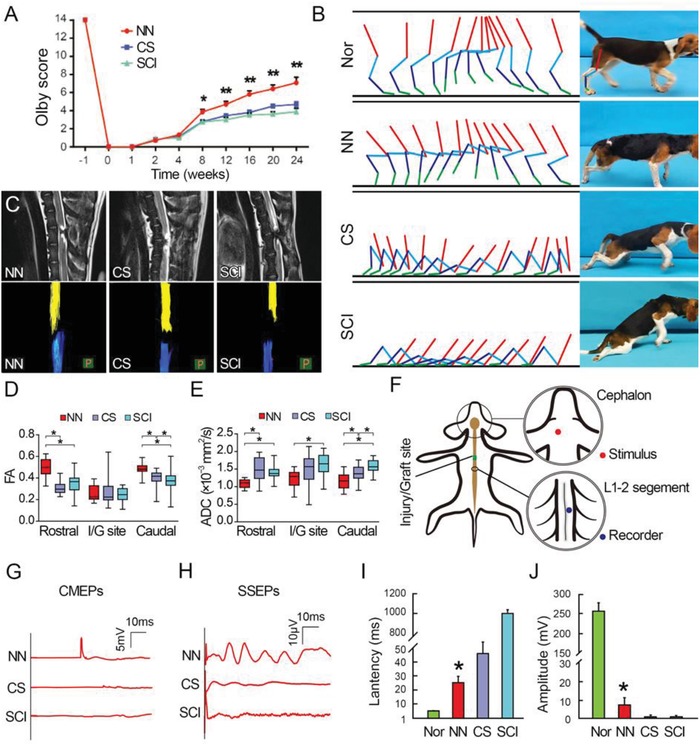
Behavior, imaging, and electrophysiological observations following NN tissue implantation. A) Gradual recovery of pelvic limb motor function was observed from the fourth week after SCI in all canines in all three groups. Olby scores in the NN group were higher than those in the CS or SCI groups 8–24 weeks after SCI. B) At 24 weeks after surgery, weight‐bearing gaits were frequently encountered in the NN group but not in the CS or SCI groups. Nor, Normal group. C) At 24 weeks after surgery, MRI confirmed complete transection injury at the T10 level in the NN, CS, and SCI groups. Canines in the NN group displayed a narrower gap between the two ends of the transected spinal cord as determined by MRI and more significant nerve tract regeneration as determined by DTI, compared with the CS and SCI groups. D) FA values in the areas rostral or caudal to the I/G site in the NN group were higher than those in the CS and SCI groups. E) ADC values in the I/G site and areas rostral or caudal in the NN group were lower than those in the CS and SCI groups. F) Schematic diagram showing the stimulation and recording sites for cortical motor evoked potentials (CMEPs). G) CMEPs were detected at the L1–L2 segments of the spinal cord in the NN group (*n* = 11), relative to negligible CMEP waves in the CS (*n* = 10) and SCI groups (*n* = 4). H) Representative spinal somatosensory evoked potentials (SSEPs) were detected in 3/11 canines in the NN group, but not in the CS and SCI groups. Bar charts for I) CMEP latency and J) amplitude showing significantly shorter latency and higher amplitude in the NN group relative to the CS and SCI groups (*p < 0.05). Nor, Normal group.

After cord transection at the T10 spinal cord segment, canines completely lost sensory and motor function below the injury level. Motor function of the pelvic limbs in the NN group (transplantation of NN tissue into the 4 mm injury gap of spinal cord, *n* = 11), CS group (implantation of CS scaffold into the 4 mm injury gap, *n* = 10), and SCI group (removal of 4 mm spinal cord tissue without any implantation, *n* = 4) was assessed using the Olby scoring scale (**Figure** **2**A,B). At 4 weeks after SCI, deep pain sensation in response to pinch was recovered in canines in all three groups (Olby score for the NN, CS, and SCI groups: 1.32 ± 0.47, 1.15 ± 0.36, and 1.00 ± 0.00). At 8 weeks after SCI, gradual recovery of pelvic limb motor function was observed in three groups. Two canines (2/11) in the NN group showed occasional multijoint swings. Pelvic limb protractions were also observed in the CS and SCI groups but with much lower frequency (Olby score for NN: 3.95 ± 0.98, CS: 2.80 ± 0.60, and SCI: 2.75 ± 0.43). Twelve weeks after SCI, pelvic limbs swings of canines in the NN group were more frequent. Two (2/11) in the NN group showed occasional weight‐bearing protractions. Six (6/10) canines in the CS group showed more than two pelvic limb joint movements but without weight‐bearing protraction. Canines in the SCI group showed mild motor function improvement in the pelvic limbs (Olby score for NN: 4.7 ± 0.95, CS: 3.45 ± 0.50, SCI: 3.00 ± 0.50). At 24 weeks after SCI, nine (9/11) canines in the NN group presented with frequent weight‐bearing gaits of the pelvic limbs for short distances (1–5 m each time, Video Clip 1 for the best performance and Video Clip 2 for average performance). In contrast, no weight‐bearing movement was observed in the CS or SCI group (Olby score for NN: 7.08 ± 1.50, CS: 4.70 ± 0.64, SCI: 3.88 ± 0.78; Video Clip 3 for the CS group and Video Clip 4 for the SCI group), consistent with observations in chronic SCI patients who have recovered mild motor function. This result suggests that transplantation of NSC‐derived NN tissue has therapeutic efficacy for restoring motor function following SCI.

To better evaluate coordinated gaits, an underwater treadmill test was used to observe locomotion in a microgravity environment (Figure S2, Supporting Information). Canines in all groups were able to regain single joint movement on the underwater treadmill 8 weeks after SCI. Locomotion recovery was not significant in the CS and SCI groups; however, pelvic limb movement in the NN group showed continuous improvement. For example, coordinated inter‐pelvic limb stepping was first observed at 12 weeks after SCI. Up to 24 weeks, frequent front‐pelvic limb coordinated stepping was observed, suggesting that NSC‐derived NN tissue may help repair the neural circuits controlling the front and pelvic limbs. Canines in the CS and SCI groups did not regain coordinated stepping even 24 weeks after SCI (Video Clip 5).

### 2.4 Imaging and Electrophysiological Assessments

Quality control of transection modeling and nerve tract regeneration was evaluated using neuroimaging. Magnetic resonance imaging (MRI) findings showed loss of spinal cord continuity at the injury site after SCI. At 24 weeks after surgery, the gap between the rostral and caudal stumps of the transected spinal cord was seen in all groups. It appeared to be narrower in the NN group compared with the CS and SCI groups (Figure 2C, top panels). Diffusion tensor imaging (DTI) was used to show tractography of the severed descending and ascending neural tracts (Figure 2C, bottom panels). At 24 weeks after SCI, DTI showed that a number of nerve bundles extended into the injury/graft (I/G) site from both the rostral and the caudal stumps in the NN group. This was in sharp contrast to the nerve fiber bundles reconstructed in the CS and SCI groups that exhibited mild regeneration into the injury site. Fiber tractography and measurement of fractional anisotropy (FA) and apparent diffusion coefficient (ADC) values are algorithms derived from MRI data to quantify nerve fiber bundle continuity. FA values in the NN group were higher in the areas rostral and caudal to the injury site compared with the CS and SCI groups (Figure 2D). In contrast, ADC values in the NN group were lower in the injury site and the areas rostral and caudal to the injury site compared with the CS and the SCI groups (Figure 2E). These results suggest that transplantation of NSC‐derived NN tissue facilitated host nerve fiber regeneration after SCI.

Electrophysiological results (Figure 2F–J) showed that canines in the NN group regained cortex motor evoked potentials (CMEPs) transmission across the I/G site to the lumbar 2 (L2) segment of the spinal cord (Figure 2F) when the motor cortex was electrically evoked. CMEP latency was significantly shorter in the NN group (25.26 ± 3.92 ms) compared with the CS (46.20 ± 7.21 ms) and SCI (46.20 ± 7.21 ms) groups (Figure 2G,I), suggesting faster electrical transmission spreading across the injury site. In addition, CMEP amplitude was evidently higher in the NN group compared with the CS and SCI groups (Figure 2G,J), suggesting that more motor pathway neurons were electrically activated. There were 3 canines (3/11) in the NN group for which spinal somatosensory evoked potentials (SSEPs) were recorded (Figure 2H). The CS and SCI groups failed to exhibit typical SSEP waves (Figure 2H). These results indicated that implanted NSC‐derived NN tissue may contribute to improved nerve conductivity in the I/G site.

### 2.5 Integration of NSC‐Derived Neurons in the Injured Spinal Cord

**Figure 3:**
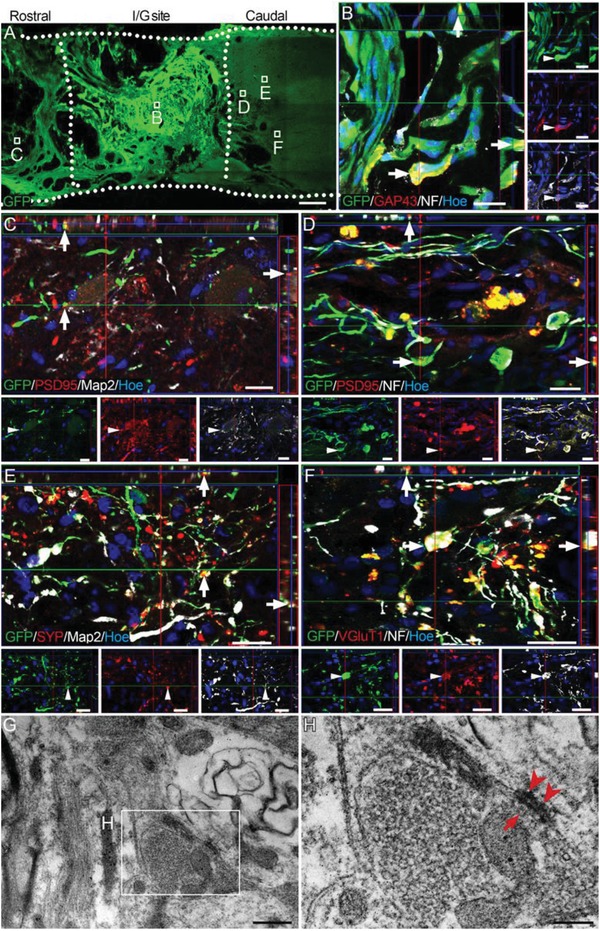
Survival and integration of NSC‐derived neurons in the injured spinal cord. A) Representative image of surviving GFP‐positive cells in the I/G site in the NN group 8 weeks after transplantation. B) GFP‐positive donor cells expressed both GAP43 and NF (arrows) in the I/G site. C–E) GFP‐positive cells extended long NF‐positive nerve fibers into the rostral and caudal areas of the I/G site, making contacts with host nerve fibers expressing the postsynaptic marker PSD95 (arrows in (C) and (D)) or expressing the presynaptic marker SYP (arrows in (E)). F) GFP‐positive nerve fibers either expressed VGluT1 or made close contact with host VGluT1‐positive fiber terminals in the area caudal to the I/G site (arrows), suggesting the presence of excitatory synapses established between donor and host neurons. Hoe, Hochest33342. G,H) TEM showed that neuronal connections in the center of the I/G site exhibited asymmetrical synapse features, as characterized by aggregation of synaptic vesicles (arrow), presynaptic components (H), and focal condensation (arrowheads) of postsynaptic membranes (H). Scale bars = 1 mm in (A); 20 µm in (B)–(F); 0.5 µm in (G); 200 nm in (H).

Eight weeks after transplantation of NN tissue, numerous GFP‐positive cells were found within the I/G site (**Figure** **3**A). A portion of the donor cells expressed growth‐related protein 43 (GAP43, Figure 3B), a marker for the growth cone of nerve fibers, suggesting dynamic axon growth. Long processes extending from donor cells in the I/G site (4 mm long) were observed in the areas rostral or caudal to the I/G site. Some processes were immunoreactive for NF (a marker for nerve fibers) or Map2, and created contacts with host neurons or nerve fibers (Figure 3C–F). Some of these contacts were immunoreactive for either PSD95 or SYP (Figure 3C–E), suggesting the presence of synaptic connections between the donor and host neurons. In addition, some donor nerve fibers extending to the area caudal to the I/G site made close contacts with host neurons that were positive for vesicular glutamate transporter 1 (VGluT1, a marker of glutamatergic neurons, Figure 3F), suggesting that excitatory synapses were established between the donor and host neurons. TEM verified the formation of synapses between neurons in the I/G site (Figure 3G,H). Asymmetrical membrane features and the presence of spherical vesicles in the presynaptic components may indicate the presence of excitatory synapses in the I/G site of spinal cord.

### 2.6 Phenotypic Characterization of NSC‐Derived Cells and Reinnervation of the I/G Site

**Figure 4:**
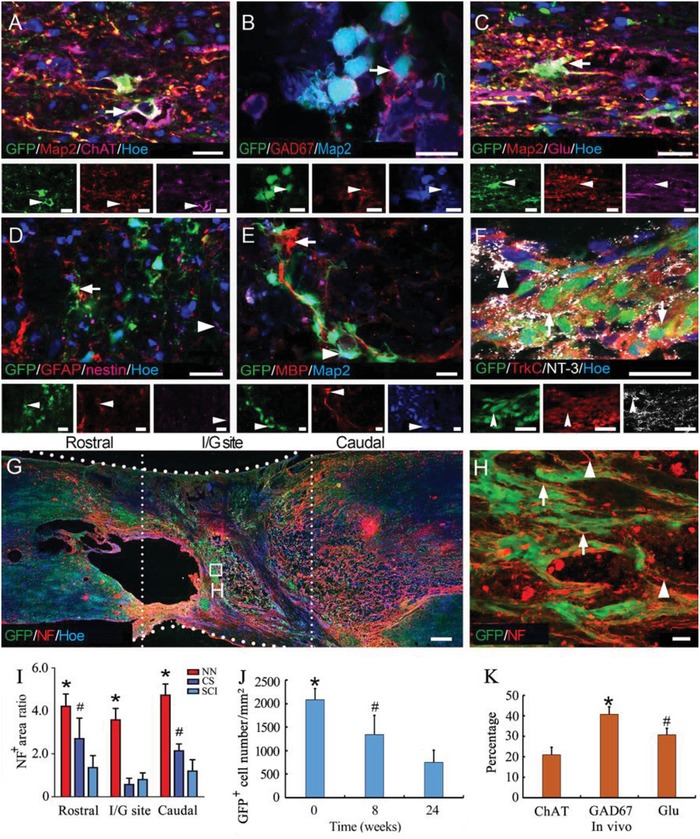
Donor cells in grafted NN tissue maintain a neuronal phenotype and contribute to enhanced innervation in the I/G site at 24 weeks. A–C) Most GFP‐positive cells were Map2‐positive neurons that expressed the neurotransmitter marker ChAT (arrow in (A)), GAD67 (arrow in (B)), or Glu (arrow in (C)). D,E) A small population of donor cells showed GFAP immunoreactivity (9.35 ± 1.76%, *n* = 5). The NSC population, as shown by nestin immunostaining (arrowhead in (D)), and oligodendrocyte population, as shown by MBP immunostaining (arrows in (E)) were negligible among GFP donor cells. F) Expression of TrkC (arrow) in GFP‐positive cells and expression of NT‐3 (arrowhead) in adjacent cells. G) Immunofluorescence staining for NF shows innervations of the I/G site and adjacent rostral and caudal areas. Hoe, Hochest33342. H) Nerve fiber outgrowths for GFP‐positive neurons (arrows) contributed to innervation in the I/G site. Host NF‐positive nerve fibers traveled longitudinally through the I/G site (arrowheads). I) Histogram showing that the NF‐positive axon density was highest in the NN group in the I/G site and areas rostral or caudal, relative to the corresponding areas in the CS or SCI groups (* and **^#^** indicate *p* < 0.05 for NN versus the CS or SCI group, respectively, *n* = 5 in the NN and CS groups, *n* = 4 in the SCI group). J) Bar chart showing the quantification of the numbers and areas of grafted GFP‐positive cells (* and **^#^** indicate *p* < 0.05 comparing 24 weeks with the 8 or 0 week data, respectively, *n* = 5 at 0 weeks, *n* = 2 at 8 weeks, *n* = 5 at 24 weeks). K) Bar chart showing the percentages of ChAT‐, GAD67‐, and Glu‐positive cells among all GFP‐positive cells in vivo at 24 weeks after transplantation (* and **^#^** indicate *p* < 0.05 when ChAT was compared with GAD67 or Glu, respectively, *n* = 5). Scale bars = 20 µm in (A)–(F) and (H), 1 mm in (G).

At 24 weeks after SCI, immunofluorescence staining was used to determine the types of cells in the NSC‐derived NN tissue in vivo. A portion of GFP‐positive cells coexpressed Map2 and ChAT or GAD67 or glutamate (Glu, a marker of glutamatergic neuron), suggesting the formation of a mixed population of excitatory and inhibitory neurons (**Figure** **4**A–C,K). In addition, a small portion of the donor cells expressed GFAP (9.35 ± 1.76%, *n* = 5). Expression of nestin (an NSC marker) or MBP was rarely detected in GFP donor cells (Figure 4D,E). In addition, there were numerous GFP‐positive cells expressing TrkC, surrounded by GFP‐negative cells expressing NT‐3 (Figure 4F).

At 24 weeks after SCI, immunofluorescence staining for NF showed that the positive area was significantly larger in the NN group (Figure 4G,H) compared with that observed in the CS and SCI groups in the I/G site and in the areas rostral or caudal to the I/G site (Figure 4I, Figure S3, Supporting Information). The nerve fibers growing out from the transplanted neurons (GFP positive) and host neurons (GFP negative), assisted in increasing innervation of the I/G site. At 8 weeks after transplantation, 60% of GFP‐positive donor cells had survived; however, at 24 weeks, this rate decreased to 35% (Figure 4J). The surviving cells at 24 weeks after transplantation were dominantly excitatory neurons (ChAT or Glu positive) rather than inhibitory neurons (GAD67 positive, Figure 4K).

### 2.7 Remyelination after Transplantation of NSC‐Derived NN Tissue

MBP immunostaining showed tube‐like immunoactive profiles wrapping the donor nerve fibers (NF and GFP positive) or host neurite in the I/G site and in rostral and caudal areas in the NN group (Figure S4A–D, Supporting Information). GFP‐positive SCs (GFP‐NT‐3‐SCs) were used to examine their potential contribution to remyelination of nerve fibers. Immunostaining showed that a large portion of GFP donor SCs were both S100 and MBP positive. SCs wrapped NF‐positive axons, suggesting that they contributed to the remyelination of nerve fibers in the I/G site (Figure S4E, E1–E3, Supporting Information). The donor SCs maintained NT‐3 expression, which may help construct a favorable microenvironment for cell survival, axon regeneration, and myelination in the I/G site of spinal cord.

### 2.8 Modification of the Microenvironment after Transplantation of NSC‐Derived NN Tissue

After transplantation of NSC‐derived NN tissue into the SCI site, the area of deposited collagenous fibers was significantly decreased as revealed by Masson's trichrome staining. In contrast, substantial deposition of collagenous fiber at the injury site was observed in the CS and SCI groups (Figure S5A, Supporting Information). The results suggested that transplantation of NSC‐derived NN tissue may help reduce the formation of fibrotic scars in the I/G site. The expression of fibronectin (FN) and laminin (LN) was not significantly different in the I/G site in the CS or SCI groups relative to the NN group (Figure S5B,C, Supporting Information). In addition, microglia/macrophages at the injury site were quantified after immunofluorescence staining of ionized calcium binding adaptor molecule 1 (IBA‐1). IBA‐1‐positive cell areas in the I/G sites and rostral or caudal to the I/G site were not significantly different among the three groups (Figure S5D, Supporting Information).

### 2.9 NSC‐Derived NN Tissue Integrated into Host Neural Circuits

**Figure 5:**
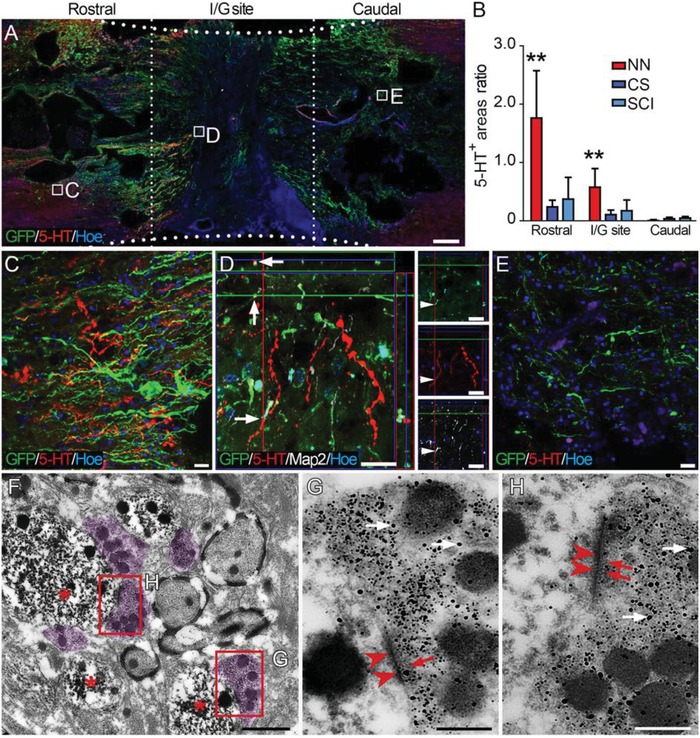
Donor neurons formed synaptic connections with descending 5‐HT‐positive nerve fibers 24 weeks after SCI. A) Overview of a longitudinal section of the spinal cord segment containing the I/G site in the NN group. Numerous donor cell processes (green) extended to the areas rostral and caudal to the I/G site, some of which were closely adjacent to 5‐HT‐positive nerve fibers (red). B) Comparison of 5‐HT‐positive areas in the I/G sites and the areas rostral or caudal in the NN, CS, and SCI groups (**p < 0.001). C) Higher magnification of the boxed area in (A) showing that 5‐HT‐positive nerve fibers were present among the dense GFP‐positive donor cell processes rostral to the I/G site. D) Some 5‐HT‐positive nerve fibers traversed into the I/G site and formed close contacts with the grafted neurons. E) 5‐HT‐positive nerve fibers were scarce in the area caudal to the I/G site. Hoe, Hochest33342. F,H) IEM showing that 5‐HT‐positive nerve fibers (labeled by nanogold particles, superimposed in light purple in (F), white arrows in (G) and (H)) formed synaptic connections with GFP‐positive donor cells (labeled by diaminobenzidine, DAB, asterisks in (F)) and resembled presynaptic components containing the vesicles as shown by nanogold particle labeling (red arrows in (G) and (H)). PSDs (arrowheads in (G) and (H)) of the donor cells were shown by DAB labeling. Scale bars = 1 mm in (A); 20 µm in (C)–(E); 1 µm in (F); 200 nm in (G) and (H).

**Figure 6:**
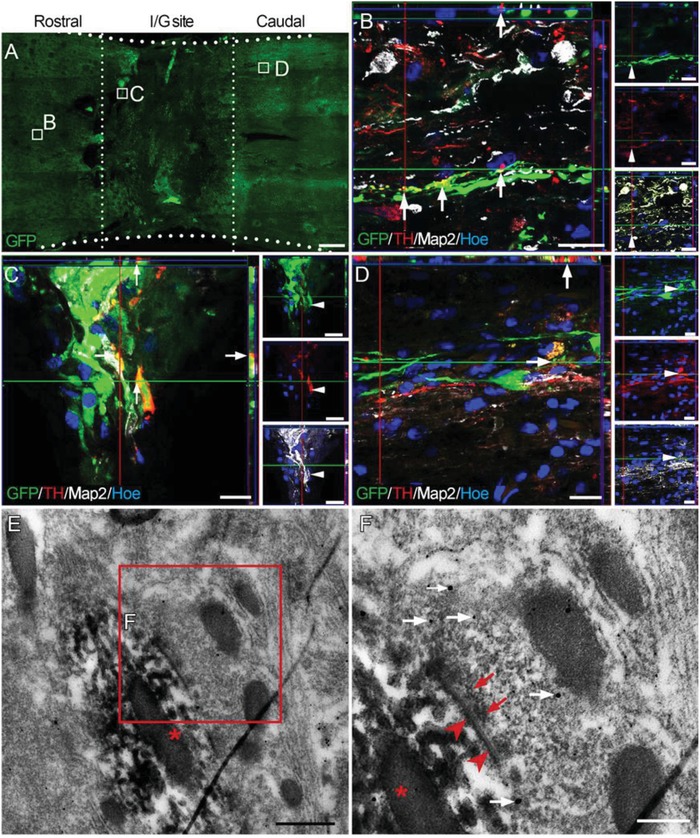
Donor neurons formed synaptic connections with descending TH‐positive nerve fibers 24 weeks after SCI. A) A horizontal section of the spinal cord containing GFP‐positive donor cells in the I/G site in the NN group. TH‐positive nerve fibers were observed B) rostral to the I/G site, C) in the I/G site, and D) in the area caudal to the I/G site. Arrows in (B)–(D) indicate where GFP‐positive donor neurons formed close contacts with TH‐positive nerve fibers. Hoe, Hochest33342. E,F) IEM showing a TH‐positive nerve fiber (labeled by nanogold particle, white arrows in (F)) formed synaptic connections with the donor cell (labeled by diaminobenzidine, DAB, asterisks). The synaptic connection had presynaptic components containing the vesicles (red arrows in (F)) and PSD (arrowheads in (F)). Scale bars = 1 mm in (A); 20 µm in (B)–(D); 1 µm in (E); 200 nm in (F).

At 24 weeks after transplantation, 5‐hydroxytryptamine (5‐HT)‐positive nerve fibers were observed rostral to the I/G site of the spinal cord in the NN group (**Figure** **5**A–C). Some 5‐HT‐positive nerve fibers grew into the I/G site and made contacts with GFP‐positive donor cells (Figure 5D). However, no 5‐HT‐positive nerve fibers were found in the area caudal to the I/G site (Figure 5E). Semi‐quantitative analysis of 5‐HT‐positive areas in the three groups suggest that the number of spared 5‐HT‐positive nerve fibers in the area rostral to the I/G site was significantly greater in the NN group compared to the other groups (Figure 5B, Figure S3, Supporting Information). Immune electron microscopy (IEM) showed synaptic connections between 5‐HT‐positive nerve fibers and GFP‐positive cells rostral to the I/G site (Figure 5F–H). Tyrosine hydroxylase (TH)‐positive nerve fibers showed more significant regeneration capability; they were present both rostral and caudal to the I/G site (**Figure** **6**A–D). TH‐positive nerve fibers also made close contacts with GFP‐positive donor cells in the I/G site and caudally (Figure 6C,D). Similarly, IEM showed that TH‐immunoreactive nerve fibers formed synaptic connections with GFP‐immunoreactive donor neurons (Figure 6E,F).

### 2.10 Structural and Functional Repair of Neural Circuits after NN Tissue Transplantation

**Figure 7:**
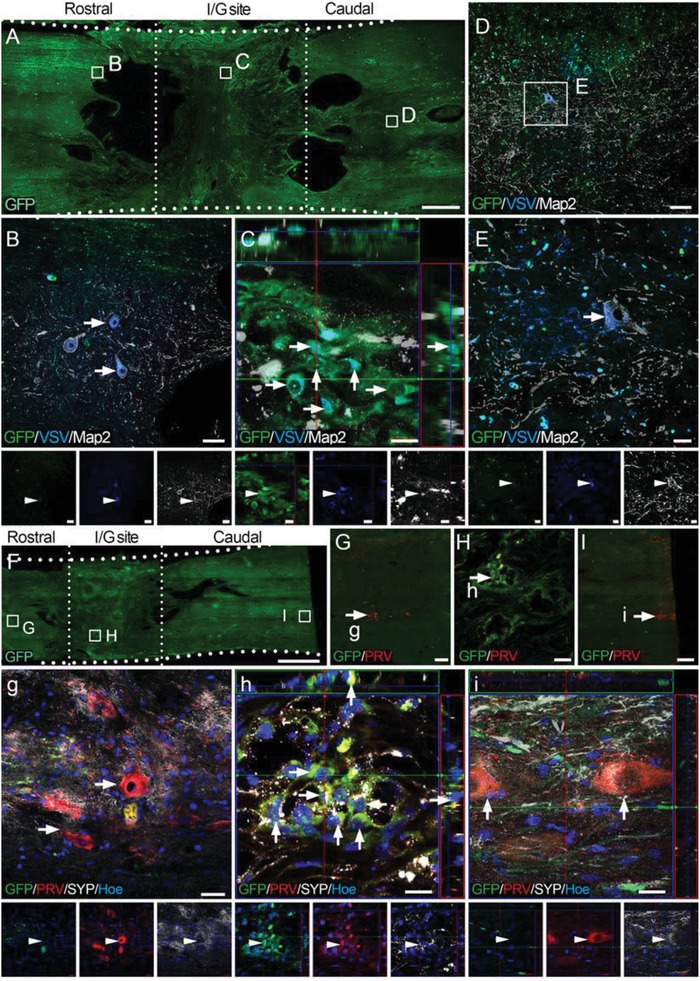
VSV anterograde and PRV retrograde tracing of transplanted NSC‐derived neurons in the I/G site. A) Low magnification of a longitudinal section from the NN group. B–D) Higher magnification of the boxed areas in (A) showing that VSV was trans‐synaptically transported from the motor cortex to neurons rostral to the I/G site (arrows in (B)) or caudal to the I/G site ((D) and arrows in (E)). VSV was clearly present in GFP and Map2 double‐positive donor neurons in the I/G site (30.12% of 249 counted GFP‐positive cells were VSV positive, arrows in (C)). F) Low magnification of horizontal spinal cord sections. G–I) Representative images showing NSC‐derived neurons (38.32% of 214 counted GFP‐positive cells were PRV positive, arrows in (H) and (h)) in the I/G site or host neurons in the areas rostral (arrows in (G) and (g)) and caudal (arrows in (I) and (i)) to the I/G site in the NN group, as shown by RFP‐PRV retrograde labeling. Hoe, Hochest33342. Scale bars = 2 mm in (A) and (F); 20 µm in (B), (H), (g), and (i); 10 µm in (C), (E), and (h); 50 µm in (D), 200 µm in (G); 500 µm in (I).

To evaluate whether disrupted neuronal circuits were repaired following NN tissue transplantation, trans‐multisynaptic viruses were used for anterograde and retrograde tracing. Vesicular stomatitis virus (VSV), an anterograde tracer,^26^ transmits across multiple synapses via the presynaptic component to the postsynaptic component. The VSV used in this study encoded blue fluorescent protein (BFP) to visualize labeled nerve tracts. The viruses were injected into the motor cortex of canines in the NN and CS groups 24 weeks after SCI. After 2 weeks, histological analysis showed no BFP fluorescence in the I/G site or caudally in the CS group (Figure S6A–C, Supporting Information). In contrast, VSV‐labeled neurons in the NN group were clearly present in the areas rostral and caudal to or in the I/G site of spinal cord (**Figure** **7**A–E). BFP fluorescence signal was observed in a large portion of GFP‐positive donor cells in the NN group (30.12% of 249 GFP‐positive cells were VSV‐positive, Figure 7C). In addition, BFP fluorescence appeared in some large neurons located in the area caudal to the I/G site (Figure 7E), suggesting that VSV transmitted across the I/G site.

Next, pseudorabies virus (PRV) was used as a retrograde trans‐multisynaptic tracer.^27^ to test whether transplanted neurons were synaptically connected with host neurons in the caudal spinal cord. In the CS group, the red fluorescent protein (RFP) signal encoded by PRV revealed host neurons several millimeters caudal to the I/G site (Figure S6D, Supporting Information), but not in the I/G site or in the area rostral to the I/G site (Figure S6E, Supporting Information). In contrast, RFP fluorescence was observed in transplanted neurons in the I/G site and in host neurons in the area rostral to the I/G site in the NN group (Figure 7F–I,g–i). Notably, 38.32% of transplanted neurons (82/214 counted GFP‐positive neurons) in the I/G site contained PRV‐RFP (Figure 7H,h). These neurons may transmit PRV to neurons in the area rostral to the I/G site of the spinal cord.

**Figure 8:**
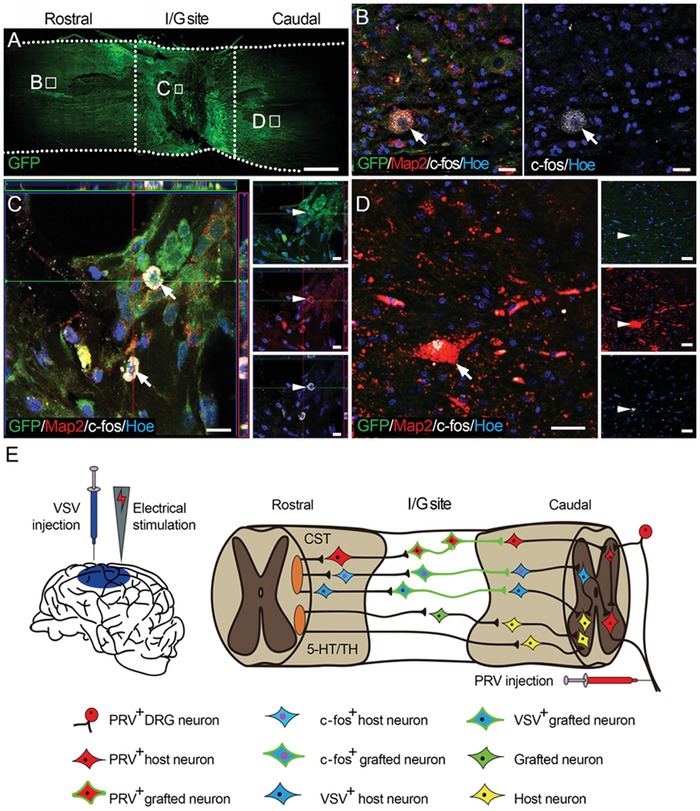
c‐fos expression in the NN group. A) Overview of a longitudinal section from the NN group. After electrical stimulation of the cerebral motor cortex, c‐fos expression was detected in the B) T7–T8 spinal cord segments, C) I/G site, and D) caudal to the I/G site. A portion of the grafted neurons were c‐fos positive (12.89% of 225 counted GFP‐positive cells, arrows in C). Hoe, Hochest33342. E) Schematic diagram depicting the putative role of the grafted NSC‐derived NN tissue in relaying motor signal as determined by anterograde transport of BFP‐VSV from the motor cortex, retrograde transport of RFP‐PRV from the sciatic nerve, and electrical stimulation of the motor cortex. Scale bars = 2 mm in (A); 20 µm in (B) and (D); 10 µm in (C).

To determine whether donor neurons were functionally integrated with host neural circuits, c‐fos immunofluorescence staining was performed after electrical stimulation of the motor cortex. Immunoreactivity was detected in host neurons rostral and caudal to the I/G site and in the transplanted neurons in the I/G site in the NN group (**Figure** **8**A–D), indicating that descending electrical signal were transmitted across multiple synapses including those established in the I/G site. Overall, 12.89% (29/225 GFP‐positive cells) of transplanted neurons expressed c‐fos (Figure 8C), suggesting that they were functionally integrated into the motor pathway. However, no c‐fos‐labeled cells were present in the I/G site or caudal to the I/G site in the CS group (Figure S6F,G, Supporting Information). These results indicate that the transplanted neurons play a pivotal role in relaying motor cortex signal across the I/G site after complete SCI (Figure 8E). To investigate long‐term survival and fate of the donor cells in the spinal cord, a canine survived up to 72 weeks after NN tissue transplantation. Histological samples show that a portion of GFP‐positive donor cells survived in the I/G site (Figure S7, Supporting Information). A subset of the donor cells maintained the phenotype of neurons (NF immunopositive, Figure S7C, Supporting Information).

## 3 Discussion

Findings from canine studies are considered to have greater translational value than those from rodent studies.^28^ Recent reports highlight the importance of validating translational programs prior to human implementation,^24^ and proof of principle in a canine model is key to this endeavor.^28^ Buoyed by success in canine SCI modeling, postoperative care, and subsequent behavioral and histological assessments, we made several important observations. First, canine NT‐3‐SCs effectively induced neuronal differentiation of TrkC‐NSCs and formation of NN tissue with functional synaptic connections. This in vitro tissue engineering approach eliminates the uncertainty of stem cell differentiation and the possibility of ectopic colony formation as seen when cells are grafted in vivo. Second, a multimodal assessment battery including locomotion scoring, MRI, DTI, and evoked potential recording showed that SCI canines with NSC‐derived NN tissue transplantation showed continuous motor and sensory improvement and were eventually able to regain coordinated weight‐bearing locomotion. Third, histological analysis showed that the transplanted NSC‐derived NN tissue created a pro‐regenerative microenvironment for donor survival and axonal regeneration. Donor neurons survived up to 24 weeks and formed synaptic connections with host neurons through long processes. Meanwhile, a significant number of host nerve fibers also regenerated into the I/G site and formed synapses with grafted neurons. Fourth, antegrade and retrograde neural tracing techniques confirmed that the transplanted neurons were successfully integrated into host neural circuits. More importantly, c‐fos in donor neurons after cortical stimulation indicated the establishment of functional synaptic connections between host cortical descending nerve fibers and donor neurons. Therefore, the NSC‐derived NN tissue integrated into existing neural circuits and functioned as a neuronal relay to restore severed connections (Figure 8E).

Unlike transplantation of embryonic spinal cord tissue^29^ or embryonic neural progenitor cells (NPCs),^30^ we preconstructed transplantable NSC‐derived NN tissue to replace lost spinal cord tissue. Our findings reinforce those from previous rat studies that NSC‐derived NN tissue was effective for structurally and functionally repairing complete SCI.^17^, ^18^ Indeed, canine NSCs and SCs, along with trophic factors and secreted ECM, formed a homeostatic microenvironment following 14 d of coculture in a cytocompatible 3D CS scaffold. Within the preconstructed NN tissue, NSCs continuously matured to achieve terminal differentiation toward multiple types of neurons, including excitatory and inhibitory neurons. These neurons were capable of firing action potentials and, more importantly, of communicating with each other through synaptic transmission, as evidenced by TEM and detection of excitatory and inhibitory postsynaptic currents in the NN tissue. SCs contributed to the formation of myelin sheathes to encapsulate neurites of NSC‐derived neurons. The NSC‐derived NN tissue demonstrated neuronal functions but was distinct from embryonic tissues, undifferentiated NSCs, or neural precursor cells (NPCs). We propose that transplantation of an NSC‐derived NN tissue may be superior to transplantation of freshly isolated NSCs or NPCs^8^, ^11^ for three reasons. 1) The homeostatic microenvironment formed during long‐duration 3D culturing conferred greater cellular resilience in the harsh post‐SCI milieu. Donor cells survived longer, allowing them to integrate with the host. 2) Transplantation of differentiated NSCs eliminated undesirable effects including excessive astrocytic differentiation and ectopic colony formation. 3) Tissue engineering in vitro allows for real‐time monitoring of the quality of each batch, thus improving the safety of stem cell therapy.

Following NN tissue transplantation in a rat model, animals regained partial motor function of the paralyzed hindlimbs. The plateau of motor recovery typically began 6 weeks after transplantation and remained stable. However, weight‐bearing hindlimb locomotion (i.e., Basso, Beattie and Bresnahan score > 9 points) was rarely observed following complete SCI in rats,^17^, ^31^ consistent with other studies using the same model. In contrast, pelvic limb motor function recovery in the complete SCI canine model following NSC‐derived NN transplantation was significantly delayed relative to rat studies. The canines started to show distinguishable motor recovery 8 weeks after NN transplantation, as assessed by Olby scoring. Significant motor recovery in the treatment group, relative to that in the controls (CS and SCI groups), was observed 12 weeks after NN transplantation. Motor function recovery in the treatment group slowed at 20 weeks and plateaued 24 weeks after transplantation. Remarkably, most canines that received NN transplantation regained weight‐bearing locomotion of the affected pelvic limbs and front‐pelvic limb coordination by the end of the study. Combined use of MRI, DTI, and electrophysiological evaluation, which is recommended as routine practice for comprehensive evaluation of SCI severity and recovery progress,^23^ also suggest that canines that received NN tissue treatment had better structural and functional improvement than those in the control groups. In contrast, canines in the control groups showed mild recovery of pelvic limb motor function with no weight‐bearing locomotion or coordinated stepping. This is similar to observations in severe SCI patients for whom spontaneous recovery of neural function is rare. Because of the biological similarities in recovery following SCI between canines and human,^24^ we hypothesize that transplantation of NSC‐derived NN tissue may also benefit SCI patients.

Given that large animals have more complex immune systems and stronger rejection to grafts than rodents,^32^ donor cell survival in vitro remains the primary challenge to stem cell‐based SCI repair.^24^ This requires immunosuppressants after cell transplantation.^3^, ^17^, ^31^ To acclimate to the drastic milieu shift, donor cells can be grafted in a trophic factor‐enriched microenvironment. For example, NSCs/NPCs encapsulated in a growth factor cocktail‐fibrin matrix had a satisfactory survival rate when transplanted into the injured monkey spinal cord.^33^ Our previous studies showed that NT‐3 overexpressing SCs promoted neuronal differentiation of NSCs in vitro^17^ and improved the microenvironment, allowing for increased graft cell survival in a rat SCI model.^18^, ^34^ In the present study, we inferred that NT‐3 overexpressing SCs may be key contributors to donor survival in the injured canine spinal cord, as we observed that NT‐3 exerted anti‐inflammatory effects when delivered to the parenchyma of the injured canine spinal cord.^35^ In addition, higher cerebrospinal fluid (CSF) pressure in the spinal cords of large animal may flush away grafted cells, preventing them from remaining and functioning at the I/G site unless intraoperative draining of CSF was performed prior to cell transplantation.^33^ However, the optimal amount of CSF draining requires further study. Moreover, draining CSF may not be a clinically acceptable strategy in humans due to numerous side effects. Alternatively, a tissue engineering approach to construct organoid or tissue‐like implants can eliminate the need to drain CSF prior to transplantation. In our study, NN tissue composed of cells, ECM, and a collagen matrix, formed a homeostatic entity following 14 d of culture. In particular, abundant ECM components including FN and LN were deposited in the scaffold. These ECM molecules enhanced attachment of seeded cells onto the CS scaffold through specific adhesion molecules such as integrins. This made resident cells more resistant to CSF pressure during implantation.

Although combined strategies have great potential, donor population loss after transplantation might be inevitable. For example, when human NPCs were grafted into nonhuman primate spinal cord for 9 months, only ≈25% of the original population remained, despite the use of a triple immunosuppressant regimen.^33^ We also observed a significant decrease in donor cell number from 8 to 24 weeks after transplantation; however, motor function continued to show improvement during this time. We speculate that this paradox may be caused by gradual functional maturation of the transplanted NN tissue and refined integration into host neural circuits. To initiate front‐pelvic limbs coordinated stepping, excitatory inputs must transmit across the grafted neurons and relay to the motor neurons controlling the pelvic limbs. It is possible that the donor neurons not functionally involved in this signaling might have been suppressed, or even eliminated. Indeed, elimination of redundant synaptic connections is essential for functional neural circuit formation.^36^ Moreover, nonintegrated neurons may have been eliminated due to a lack of survival signals from other neurons.^37^ Future work is needed to distinguish whether neuronal death following transplant is a result of neural circuit maturation or lack of transplanted tissue viability.^38^ Nonetheless, there were still GFP positive donor cells survived in the I/G site of spinal cord up to 72 weeks after NN transplantation. It would be worthy to systematically analysis the biological basis supporting the long‐term survival of the donor neurons in the future.

A pro‐regenerative microenvironment during transplantation of NSC‐derived NN tissue may also promote endogenous neural regeneration. For example, the fibrotic scarring represented by collagen deposition was significantly reduced in the NN group. Conversely, the expression of FN and LN was upregulated in the I/G site of the NN group. The shift between the inhibitory and pro‐regenerative ECM molecules may also promote regeneration of host nerve fibers and endogenous neurogenesis in the I/G site. The canines in the NN group showed continuous motor function recovery; therefore, endogenous neural regeneration may compensate for the gradual loss of donor neurons. However, further research is warranted to determine the delicate interactions between endogenous neural regeneration and exogenous neurons.

Histological analysis was performed to show how NSC‐derived NN tissue contributed to structural repair of the damaged canine spinal cord. At 8 weeks after transplantation, most GFP‐positive donor neurons located at the I/G site had long processes extending rostral or caudal to the I/G site. Synaptic connections between the donor and host neurons were verified by immunocytochemistry and IEM. It was inferred that NT‐3 activated TrkC signaling to promote synapse formation^18^, ^39^ and that TrkC‐overexpressing neurons more readily formed synaptic connections with host‐regenerated nerve fibers expressing protein tyrosine phosphatase σ (PTPσ).^40^ However, we did not observe long axons that extended to remote spinal cord tissue more than two spinal cord segments rostral or caudal to the I/G site. In other words, there were no direct synaptic connections between the donor neurons and lumbar motor neurons controlling pelvic limb muscles. However, trans‐synaptic virus tracing demonstrated establishment of multisynaptic connections in the motor neural pathway. VSV, a type of anterograde trans‐multiple synaptic virus,^26^ transmitted from the motor cortex (injection site) to the transplanted neurons in the I/G site, as evidenced by BFP fluorescence encoded by VSV in GFP/Map2 double‐immunopositive donor neurons. BFP was detected in host neurons in the area caudal to the I/G site, meaning that VSV was transmitted from the donor neurons in the I/G site to the host neurons caudal to the I/G site through established synapses. No BFP was detected in lumbar neurons, which might be due to insufficient time after injection to allow the virus to transmit. However the retrograde trans‐multiple synaptic virus PRV transmitted from the lumbar motor neurons to neurons in the area caudal to the I/G site, where they retrogradely transmitted to the implanted neurons in the I/G site. In contrast, no VSV or PRV transmission was detected in the I/G site of canines in the CS group. These results indicated that the transplanted NN tissue contributed to repair of the severed spinal cord with a large tissue deficit. Although our previous publication suggested that MSC‐derived NN tissue may also contribute to structural repair of canine injured spinal cord,^16^ this study included de facto synaptic connection mapping of the key elements in the motor pathway starting from the cortex to lumbar motor neurons.

It is plausible that transplanted NSC‐derived NN tissue may relay excitatory signals across the I/G site, contributing to recovery of paralyzed motor function after SCI. This is supported by the observations from this study. 1) Canines transplanted with NSC‐derived NN tissue regained partial motor function of the pelvic limbs, as evidenced by recovery of weight‐bearing locomotion and coordinated stepping on an underwater treadmill, relative to mild motor recovery in canines in the control groups. 2) CMEP recordings indicated that electrical signals initiated from the motor cortex could pass through the I/G site to reach the lumbar spinal cord. 3) After electrical stimulation of the cerebral cortex, c‐fos expression was found in neurons rostral to the I/G site, in grafted neurons, and in neurons caudal to the I/G site. This suggests that action potentials were conducted through functional synapses connecting the grafted and host neurons.^41^ 4) Histological analysis further verified that host descending 5‐HT‐ and TH‐positive nerve fibers, both of which conduct excitatory signals from the brain, were able to regenerate significantly to the rostral and central areas of the I/G site to form synaptic connections with the transplanted neurons. 5) More than half of the donor neurons were capable of synthesizing excitatory neurotransmitters. Axons of donor neurons extended to the adjacent caudal spinal segment of the host tissue and made synaptic connections with the host neurons via VGluT1‐positive presynaptic boutons. The PSD was immunopositive for PSD95, a marker for excitatory postsynaptic components. Asymmetric synapse features observed by IEM further suggest that excitatory synapses were established between donor and host neurons. Taken together, these results indicate that the implanted NN tissue played an essential role in re‐establishing functional connections in the motor pathway, allowing for excitatory transmission from the brain to the muscle, which may explain how weight‐bearing locomotion and coordinated stepping were regained. Indeed, reactivating the spared motor pathway is believed to directly control motor function. For example, epidural stimulation of lumbar motor circuits improves motor recovery in SCI patients.^42^, ^43^ In contrast, suppressing overinhibition caused by inhibitory interneuron activity following SCI was shown to promote functional recovery in a mouse SCI model.^44^ Sensory recovery is considered fundamental for motor recovery. Front‐pelvic limb coordinated stepping on the underwater treadmill, as observed in this study, may indicate that canines with NN tissue transplantation had at least partial proprioception. Validation of sensory recovery in animals requires special equipment (e.g., functional MRI) or sophisticated training and compliance to the test, which were not performed in this study. However, SSEP detection indicated that the implanted NN tissue may have helped relay the ascending sensory signals. This is supported by the fact that the ascending sensory pathway helps the cortex adjust the accuracy of the descending motor pathway, which is essential for recovery of coordinated movements.^45^ Furthermore, SC‐derived myelin may contribute to functional recovery after SCI as remyelination enables more stable and efficient neuronal relay by NN tissue.^46^

## 4 Conclusions

Functional NN tissue was successfully constructed in a 3D culture system by coculturing canine NT‐3‐SCs and canine TrkC‐NSCs in a CS scaffold. These NN tissues survived up to 72 weeks at the SCI site, served as interneurons to relay descending excitatory signals from the brain to host neurons caudal to the I/G site, and helped canines regain weight‐bearing locomotion and coordinated stepping. Adverse effects such as autotomy, hyperalgesia, and tetanic spasm were not encountered following transplantation. Histological analysis ruled out ectopic migration or tumorigenesis by donor cells. These findings support the safety and efficacy of transplantation of NN tissue to treat SCI in large animals and provide a framework for future clinical translation using tissue engineering construction of NSC‐derived NN tissue. Using induced pluripotent stem cell‐derived NPCs^6^, ^7^ or SCs to construct NN tissue may be a future strategy to mitigate cell source concerns. Combination with physiotherapy or rehabilitation^47^, ^48^ may enhance integration of the transplanted NN tissue with donor neural circuits, which could further increase neural function restoration following SCI.

## 5 Experimental Section

*Ethics*: All animal experiments were approved by the Institutional Animal Care and Use Committee of Sun Yat‐sen University (Approval number: 20181000244), and the Laboratory Animal Regulations of Guangdong Province (2010 No. 41). Animal welfare was in compliance with Laboratory Animal Guidelines for Ethical Review of Animal Welfare, General Administration of Quality Supervision, Inspection and Quarantine of the People's Republic of China/Standardization Administration of China (GB/T 35892‐2018).

*Cultivation of NSCs and SCs*: To acquire NSCs and SCs, newborn male beagle canines (1–3 d, ChaiMen Biological Inc., Nanjing, China) were anesthetized and sacrificed by cervical dislocation after inhalation of overdosed isoflurane. NSCs were isolated from the hippocampus similar to a previously described procedure.^49^ Briefly, the whole hippocampus was dissected and dissociated into single cell suspension. NSC medium composed of Dulbecco's minimum essential medium (DMEM)/F12 (1:1, Life Technologies, USA) supplemented with 1 × B27 and 20 ng mL^−1^ bFGF. Cells were grown as neurospheres in suspension and passaged by mechanical dissociation every 5 d. Neurosphere progenitor content was assessed by nestin immunostaining (Figure S1A, Supporting Information).

To obtain SCs, the sciatic nerves and brachial plexus were dissected and placed in ice‐cold D‐Hank's solution. The epineurium and connective tissue were removed under a dissecting microscope. All nerves were cut into small pieces (< 2 mm) and dissociated with 0.16% collagenase (Sigma‐Aldrich, USA) at 37 °C for 15 min and centrifuged at 1000 rpm min^−1^ for 5 min. Pellets were resuspended in 1 mL culture medium containing DMEM/F12, 10% fetal bovine serum (2 mmol L^−1^ forskolin (Sigma‐Aldrich, USA)) and 20 mg mL^−1^ bovine pituitary extract (Sigma‐Aldrich, USA), then seeded in 75 mL culture flasks precoated with polylysine at 37 °C with 5% CO_2_. After 30 min, 4 mL of culture medium was added to each flask. Cells were maintained in an incubator at 37 °C in 5% CO_2_. Culture medium was changed every 2 d. The cells were passaged at 90% confluence and purified by differential adhesion and differential digestion techniques.^16^ SC purity was assessed by immunochemical staining with S100.

*In Vitro Induction of NN Tissue*: Recombinant lentiviruses were used to modify NSCs and SCs. Neurospheres were transfected with a lentivirus carrying a TrkC coding sequence (pLent‐EF1a‐TrkC‐Flag‐CMV‐GFP‐P2A‐Puro) (Vigenebio Biosciences Inc., China); SCs were infected with a lentivirus vector carrying an NT‐3 sequence (pLent‐EF1a‐NT‐3‐Flag‐CMV‐P2A‐Puro or pLent‐EF1a‐NT‐3‐Flag‐CMV‐GFP‐P2A‐Puro) (Vigenebio Biosciences Inc.). After the lentivirus was added into the culture medium at a multiplicity of infection of 50 for 48 h, the supernatant was removed and replaced with fresh culture medium containing 2 µg mL^−1^ puromycin for cell purification. The screened TrkC gene‐modified NSCs (TrkC‐NSCs) and NT‐3 gene‐modified SCs (NT‐3‐SCs) were mixed in a 1:1 ratio with 1 × 10^6^ cells seeded into a cylindrical CS scaffold (5 mm diameter and 4 mm long) using a micropipette. The scaffolds were incubated for 14 d, and the culture medium was changed every day. The experimental groups included NSCs, NSCs+SCs, T‐NSCs+SCs, NSCs+N‐SCs, and T‐NSCs+N‐SCs, with a total of 1 × 10^6^ cells (for NSCs alone or 1:1 for NSCs and SCs coculturing) in 30 µL of culture medium seeded into each CS.

*Western Blotting*: Scaffolds in each group (*n* = 5) were chopped into pieces on a cold stage, added to radioimmunoprecipitation assay buffer, and sonicated to extract total protein. Equal amounts of protein in each group were loaded onto a 10% polyacrylamide gels for electrophoresis. After transfer to polyvinylidene fluoride membranes, the membranes were treated with the following primary antibodies: NF, TrkC, PSD95, ChAT, GAD67, GFAP, SYP, MBP, NT‐3, and glyceraldehyde‐3‐phosphate dehydrogenase (GAPDH) (all antibodies are from rabbit host species) and incubated overnight at 4 °C. Membranes were then incubated with antirabbit horseradish peroxidase‐conjugated IgG. Bands were detected with an enhanced chemiluminescence western blot kit (Cwbiotech, China) using a chemiluminescence imaging system (ChemiDoc, Bio‐Rad, USA). GAPDH was used as a loading control. The antibodies used in this study are listed in Table S1 (Supporting Information).

*Immunofluorescence Staining*: Expression of specific proteins was detected by immunofluorescence. Briefly, spinal cord tissue was cut into 25 µm thick longitudinal sections using a cryostat microtome, then rinsed with 0.01 m phosphate‐buffered saline (PBS) three times, blocked with 10% goat serum for 30 min, and incubated with primary antibody containing 0.3% Triton X‐100 to increase penetration at 4 °C overnight. The sections were washed three times with PBS and then incubated with a secondary antibody at 37 °C for 1 h, Hoechst33342 (Hoe) was used to stain the nuclei. The sections were observed with a fluorescence microscope (Leica, Germany) or a laser confocal microscope (LSM780/LSM800, Zeiss, Germany) to produce a *z*‐axis scan. The list of antibodies used is shown in Table S1 (Supporting Information).

*Live‐Cell FM1‐43 and Whole Cell Patch‐Clamp Detection*: FM1‐43 [*N*‐3‐triethylammonmpropyl)‐4‐(4‐(dibutylamino) styryl] dye (Life Technologies) was used to determine whether induced neurons had synaptic vesicle releasing features.^50^ After 14 d of coculture, the NN tissue was rinsed with low [K^+^] 4‐(2‐hydroxyethyl)‐1‐piperazineethanesulfonic acid (HEPES). The first dose of high [K^+^] solution stimulated recycling of FM1‐43‐containing endocytic synaptic vesicles. After rinsing three times (15–20 min each) with culture medium in the absence of FM1‐43, endocytic/exocytotic activities decreased to basal levels, and nonspecific labeling of cytoplasmic membranes was also eliminated, while synaptic vesicles maintained FM1‐43 labeling. After rinsing, cells were excited with a second dose of high [K^+^] solution without FM1‐43, resulting in depolarization. FM1‐43‐labeled synaptic vesicle release was visualized using a confocal laser scanning microscope (LSM780/LSM800, Zeiss, Germany). A control experiment to assess nonspecific bleaching of fluorescence was performed simultaneously on NN tissue without high [K^+^] solution stimulation.

To investigate excitability of NSC‐derived neurons, whole‐cell patch clamp was performed with a HEKA EPC amplifier 10 (HEKA Inc., Germany) after culturing for 21 d in vitro. Results were analyzed using Patchmaster software (HEKA Inc.). Signals were filtered at 1 kHz and sampled at 5 kHz. The external solution contained 140 × 10^−3^ m NaCl, 5 × 10^−3^ m KCl, 2 × 10^−3^ m CaCl_2_, 1 × 10^−3^ m MgCl_2_, 10 × 10^−3^ m HEPES, and 10 × 10^−3^ m glucose (320 mOsm, pH set to 7.3 with Tris base). The patch electrodes had a resistance of 3–5 MΩ when filled with pipette solution containing 140 × 10^−3^ m CsCl, 2 × 10^−3^ m MgCl_2_, 4 × 10^−3^ m ethylene glycol‐bis(β‐aminoethyl ether)‐*N*,*N*,*N*′,*N*′‐tetraacetic acid (EGTA), 0.4 × 10^−3^ m CaCl_2_, 10 × 10^−3^ m HEPES, 2 M magnesium adenosine triphosphate (Mg‐ATP), and 0.1 × 10^−3^ m guanosine triphosphate (GTP). The pH was adjusted to 7.2 with Tris base, and the osmolarity was adjusted to 280–300 mOsm with sucrose. Briefly, when the micropipettes were at the appropriate distance from the cell membrane, brief and gentle suction was applied to create tight contact with resistance up to 1 GΩ. Then, brief and strong suction was used to form a whole‐cell configuration with tip resistance of 3–5 MΩ. Electrophysiological recordings were performed at room temperature (22–24 °C). Finally, the membrane potential of the cells was clamped at −70 mV using a voltage clamp. mPSCs were counted and analyzed using Fitmaster (HEKA Inc.).

*Spinal Cord Transection Modeling and Transplantation*: Twenty‐seven healthy female beagles (6–8 months old, 8–9 kg, supplied by Frontier Biotechnology Inc., China) were randomly divided into three groups: 1) the NN group (*n* = 11 for 24 weeks, *n* = 2 for 8 weeks, *n* = 1 for 72 weeks); 2) the CS group (*n* = 10 for 24 weeks); and 3) the SCI group (*n* = 4 for 24 weeks). To ensure adherence to inclusion criteria, all animals were screened for signs of spinal cord disorder prior to the experiment.

Spinal cord transection was performed as previously described.^16^ Briefly, canines were anesthetized, and the spine was cut at thoracic vertebrate T10. Laminectomy was performed to provide a window for a longitudinal dura incision of about 1 cm. After the T9‐T10 spinal segments were exposed, a sharp blade was used to transect the rostral and caudal spinal cord to make a 4 mm long gap. The severed spinal cord was removed until the ventral dura was exposed to ensure that no residual spinal cord tissue remained in the gap. The corresponding paired spinal roots were also completely removed. The NN or CS scaffold was implanted after sufficient hemostasis in the NN and CS animals, respectively. No scaffold was implanted in the SCI group, but the area was washed three times with saline. Low‐tension dural sutures were placed to prevent scaffold mobility. Intensive postoperative care was administered during the first week, including daily intravenous rehydration with 50 mL lactated Ringer's solution. Penicillin was administered to prevent infection (800 000 units, intramuscular daily). Pain was managed by oral administration of meloxicam (0.1 mg kg^−1^/24 h for 5 d). Defecation was stimulated manually three times per day until bladder function returned. Cyclosporin A (20 mg kg^−1^) was administered once daily on a strict 24 h cycle until the end of the experiment.

*Assessment of Locomotor Performance*: The behavioral assessment battery included an open field locomotion test platform equipped with a high‐speed videotaping device and an underwater treadmill locomotion evaluation system. Motor function recovery assessment was performed by a double‐blind protocol using Olby scoring.^51^, ^52^ During evaluation, canines were free to walk in the open field for more than 10 min. Scores were obtained based on limb movement functions including deep pain reflex, joint movement, weight‐bearing capability, muscle strength, and gait on a 15‐point scale. To further assess pelvic limb locomotion under non‐weight‐bearing conditions, canines were placed on an underwater treadmill for visual observation of minor joint movement and coordinated movements between the front and contralateral pelvic limbs, and between the pelvic limbs.^16^ Canines were placed on a treadmill submerged in warm water (35–39 °C). Track belt speed was adjusted according to individual maximum values (5–20) cm s^−1^ and the duration for each canine on the treadmill was 5 min for each recording cycle. Two investigators blinded to the experimental groups scored the videos captured at 0, 1, 2, 4, 8, 16, and 24 weeks postoperatively.

*Electrophysiology Assessment*: CMEPs and SSEPs were assessed at 24 weeks after SCI using NeuroExam M‐800 (Medocon Technology, China). Canines were anesthetized with pentobarbital sodium (3% dissolved in saline, 45 mg/kg, intraperitoneal [i.p.]) and ketamine (10 mg kg^−1^, intramuscular injection every 20 min during surgery), and placed in a prone position during the operation. For CMEPs, one stimulus electrode was inserted into the subcutaneous tissue 3 cm lateral to the intersection of the cranial midline and eyebrow, and another electrode was inserted into the contralateral deep tissue approaching the bone surface between the rear ear and inion. The recording electrode was placed on the spinal cord surface at L1–L2. The grounding electrode was inserted subcutaneously near the T1 spinal segment. Multiple pulse stimulation was used to elicit a CMEP with the following parameters: 250 times gain, 150 µs time constant, 100 mA pulse width, and 1000 µs interval repeated four times. Each test was repeated 40 times to ensure waveform stability. For SSEPs, the recording electrodes were placed in the same location as the stimulus electrode for CMEPs, and the stimulus electrode was placed at the gluteus maximus. A series of stimulations with 7 mV, 1 Hz, and 300 repeats was performed for SSEP waveform stacking.

*Induction of Spinal Cord c‐fos Expression*: At 24 weeks after SCI, canines in the NN group (*n =* 2) and the CS group (*n =* 2) received unilateral electrical stimulation of the motor cortex to induce c‐fos expression in the spinal cord. Canines were anesthetized with 1% pentobarbital sodium (40 mg kg^−1^, i.p.), and the motor cortex zone was exposed by craniotomy. Stimulation was delivered between an electrode (0.7 mm diameter) on one side of the hindlimb motor cortex (1.0–1.2 cm posterior to the bregma, 1.0 cm lateral to the midline) and a needle electrode on the hard palate. A 300 ms train current stimulation was delivered at 10 mA with a pulse duration of 0.5 ms at a frequency of 200 Hz continuously for 1 h.^16^, ^41^ Then, the canines remained deeply anesthetized for 2 h until sacrifice.

*Trans‐Multisynaptic Virus Labeling*: At 24 weeks after SCI, two canines per group were randomly selected from the NN and CS groups and received VSV (BrainVTA Technology Co. Ltd., China) to label the motor cortex for anterograde virus mapping. The labeling procedure was performed as previously described.^16^, ^53^ Briefly, after anesthetization, the head of the canine was fixed in a custom‐made stereotactic frame. A longitudinal incision (6–7 cm) was made at the midline above the parietal bone. To expose the motor cortex, two 1.5 × 3.0 cm^2^ oval windows were made in the parietal bone using a drill and a pair of rongeurs with the following coordinates: 1.0–1.2 cm posterior to the bregma, 1.0 cm lateral to the midline. VSV‐encoding BFP (2.00E+09 PFU mL^−1^) was loaded into a Hamilton microsyringe (Hamilton Co., USA) attached to the stereotactic frame. A total of ten injections (3 µL each) were made in both cerebral hemispheres, delivering a total of 30 µL VSV.

For retrograde virus mapping, two canines per group were randomly selected from the NN and CS groups and underwent retrograde PRV (BrainVTA Technology Co. Ltd.) labeling at the sciatic nerve. Briefly, after anesthetization, a 5 cm longitudinal incision was made along the biceps femoris and semitendinosus muscles of both pelvic limbs to expose the sciatic nerve as much as possible. With the aid of a dissecting stereomicroscope (Leica), the needle tip of a 30 G Hamilton syringe was inserted into sciatic nerve along its longitude axis for 10 mm, and then withdrawn 2 mm to make space for injection. PSV (20 µL, 2.00E+09 PFU mL^−1^) which encoded the RFP reporter gene (RFP‐PRV) was slowly injected into the bilateral sciatic nerves. After injection, the sciatic nerves were clamped with hemostatic forceps 2 cm above the injection point for 30 s to maximize PRV particle uptake by the nervous tissue, and the wound was sutured layer by layer.

After virus mapping, the canines received extensive postoperative care including intramuscular injection of penicillin (80000 U/kg/d) for 3 d to prevent infection. The animals were sacrificed 14 d after virus injection.

*MRI and DTI*: The canines were examined by MRI and DTI 24 weeks after surgery to observe the morphology of the spinal cord lesion site. Fiber tractography and measurement of FA and ADC values were acquired to assess the continuity of nerve fiber bundles in the spinal cord, which is not detectable by MRI.^54^, ^55^ The average FA and ADC values were measured and represent the rostral, caudal, and I/G site of the spinal cord for each animal.

*Ultrastructural Observation*: Tissue engineering NN tissue was visualized by SEM. Samples were washed three times with PBS, then fixed with 2.5% glutaraldehyde for 90 min before dehydration using an alcohol gradient. After freeze‐drying for 2 d, the samples were coated with gold, and then observed by SEM (Philips XL30 FEG, Philips, Netherlands).

TEM was used to observe the NN tissue or spinal cord tissue. The samples were fixed with 2.5% glutaraldehyde and 15% picric acid for 2 h at 4 °C, followed by 1% osmic acid for 1 h at room temperature, dehydrated using an alcohol and acetone gradient, then embedded in Epon and polymerized for 48 h at 60 °C. The embedded tissue was sliced into semithin sections (2 µm thickness, Leica RM2065 microtome). Sections were mounted on slides, stained with toluidine blue (5% in a borax solution), and mounted using neutral balsam prior to observation. The remaining contents were cut into ultrathin sections (100 nm thickness), double‐stained with lead citrate and uranyl acetate, and examined using an electron microscope (Philips CM 10, Philips, Netherlands).

For IEM, the injured spinal cord segment was removed and immediately immersed in 4% paraformaldehyde containing 0.15% glutaraldehyde and 15% saturated picric acid at 4 °C for 2 h. After rinsing in cold 0.1 M PBS, the tissues were fixed without glutaraldehyde at 4 °C for 4 h, then sagittally sectioned into 50 µm thick slices using a vibratome. Sections were transferred to 0.1 m PBS containing 25% sucrose and 10% glycerol overnight at 4 °C, then freeze‐thawed three times using liquid nitrogen. For double‐labeling, the tissue slices were treated with 0.3% H_2_O_2_ to scavenge endogenous peroxidase prior to adding blocking serum. After washing with PBS, sections were treated with 20% goat serum (Tris buffer, pH 7.4) for 40 min prior to incubation with primary antibodies. The sections were incubated with primary antibodies in 2% goat serum for 24 h at 4 °C, then incubated with secondary antibody overnight at 4 °C. The tissues were postfixed with 1% glutaraldehyde for 10 min and further treated using an SABC‐DAB Kit and gold enhanced using a GoldEnhance EM Plus Kit (NanoProbe 2114, USA). The sections were then subjected to osmiumization, gradient dehydration, then embedded with Epon. Finally, ultrathin sections were observed using the electron microscope.

*Morphological Quantification*: For the in vitro quantification of immunopositive cells, one in every five of the whole series of horizontal sections (five sections) from each NN tissue was selected (*n* = 5 per group). After immunostaining with the respective markers, five areas (0.7 mm × 0.5 mm including four corners and one center) were chosen for each of the sections. The percentage of immunopositive cells was calculated by counting the total number of immunopositive cells and dividing it by the total number of GFP positive cells.

For the quantification of surviving cells, prior to transplantation (0 weeks), one in every 10 of the whole series of horizontal sections (five sections) from each in vitro cultured NN tissue was selected (*n* = 5). Five areas (0.7 mm × 0.5 mm including four corners and one center) were chosen for each of the sections, and the total numbers of GFP‐positive cells was counted. The numerical value obtained was subsequently divided by the total area. For the quantification of surviving cells at 8 weeks (*n* = 2) and at 24 weeks (*n* = 5), one in every ten slices of the whole series of horizontal sections (five sections) from each animal was selected. The transected area with implantation was defined as the I/G site. Five areas (0.7 mm × 0.5 mm including four corners and one center) for each section were chosen in the I/G site. The total number of GFP‐positive cells was counted and divided by the total area.

For the in vivo quantification of immunopositive cells, areas in the I/G site of each of the horizontal sections were scrutinized. One in every ten sections from each animal was processed, and a total of five sections per animal were analyzed (*n* = 5). Five areas (0.7 mm × 0.5 mm including four corners and one center) for each of the sections cut through the I/G site were chosen. The percentage of immunopositive cells was calculated by counting the total number of immunopositive cells and dividing by the total number of GFP‐positive cells.

To investigate nerve fiber density, one in every five slices of the whole series of sections from each animal was selected, and the entire areas of each slice containing T8–T10 spinal cord segments were visualized using a laser confocal fluorescence microscope after immunofluorescence staining. A total of 10 sections per animal were analyzed (*n =* 5 for the NN and CS groups respectively, *n =* 4 for the SCI group). The range of rostral or caudal areas was defined as a 0.5 mm wide area rostral or caudal to the transection margin, respectively. The transected area with or without implantation was defined as the I/G site. Three areas (325 µm × 325 µm) were selected as regions of interest (ROIs) from each photographed slice. These ROIs were processed using ImageJ software (version 1.48, NIH, USA) and the positive area ratio was calculated. Images cavity areas > 50% were excluded. This method was utilized for the semi‐quantitative analysis of NF‐, 5‐HT‐, FN‐, LN‐, and IBA‐1‐positive areas.

*Statistical Analysis*: All statistical analyses were performed using SPSS 17 (SPSS Inc., USA). Data were expressed as the means ± standard deviations (mean ± SD). Data were analyzed using one‐way analysis of variance (ANOVA). If equal variances were found, the least‐significant difference (LSD) test was applied. If variances were not equal, Kruskal‐Wallis test and Dunnett's T3 were applied. Student's *t*‐test was used to compare two groups. *p* < 0.05 was considered statistically significant.

## Conflict of Interest

The authors declare no conflict of interest.

## Supporting information

Supplementary

Supplementary

Supplementary

Supplementary

Supplementary

Supplementary
