# Supplementary material for: Tissue‐Engineered Neural Network Graft Relays Excitatory Signal in the Completely Transected Canine Spinal Cord
Source: Adv Sci (Weinh). 2019 Sep 19;6(22):1901240. doi: 10.1002/advs.201901240 (PMC6864506; doi:10.1002/advs.201901240)
Supplement: Supplementary file 1 — Supplementary [file ADVS-6-1901240-s002.pdf]

Copyright WILEY-VCH Verlag GmbH & Co. KGaA, 69469 Weinheim, Germany, 2019.

ADVANCED SCIENCE
Open Access

Supporting Information

for *Adv. Sci.,* DOI: 10.1002/advs.201901240

Tissue-Engineered Neural Network Graft Relays Excitatory Signal in the Completely Transected Canine Spinal Cord

Bi-Qin Lai, Ming-Tian Che, Bo Feng, Yu-Rong Bai, Ge Li, Yuan-Huan Ma, Lai-Jian Wang, Meng-Yao Huang, Ya-Qiong Wang, Bin Jiang, Ying Ding, Xiang Zeng, and Yuan-Shan Zeng**

Supporting Information

Tissue-Engineered Neural Network Graft Relays Excitatory Signal in the Completely Transected Canine Spinal Cord

Bi-Qin Lai#, Ming-Tian Che#, Bo Feng, Yu-Rong Bai, Ge Li, Yuan-Huan Ma, Lai-Jian Wang, Meng-Yao Huang, Ya-Qiong Wang, Bin Jiang, Ying Ding, Xiang Zeng, Yuan-Shan Zeng**

Table S1 Primary and secondary antibodies

Antibodies	Species	Type	Dilution	Source (Catalog)
Neurofilament 200 (NF)	Mouse	Monoclonal IgG	1:1000	Sigma–Aldrich, St. Louis, USA (N0142)
Neurofilament 200 (NF)	Rabbit	Polyclonal IgG	1:400	Merck Millipore, Billerica, USA (N4142)
Synaptophysin (SYP)	Mouse	Monoclonal IgG	1:200	Sigma–Aldrich, St. Louis, USA (S5768)
Postsynaptic Density Protein 95 (PSD95)	Rabbit	Polyclonal IgG	1:800	Abcam, London, UK (ab18258)
CaMKII alpha (phospho T286)	Rabbit	Polyclonal IgG	1:500	Abcam, London, UK (ab5683)
Microtubule-associated protein 2 (Map2)	Mouse	Monoclonal IgG	1:1000	Sigma–Aldrich, St. Louis, USA (M4403)
Choline Acetyltransferase (ChAT)	Rabbit	Polyclonal IgG	1:800	Merck Millipore, Billerica, USA (AB2219)
Glutamate (Glu)	Rabbit	Polyclonal IgG	1:500	Boster, Wuhan, China (BA0604-1)
Glutamic acid decarboxylase 1 (GAD67)	Rabbit	Monoclonal IgG	1:500	Abcam, London, UK (ab213508)
TrkC	Goat	Polyclonal IgG	1:300	Sigma–Aldrich, St. Louis, USA (T2450)
Neurotrophin-3 (NT-3)	Rabbit	Polyclonal IgG	1:300	Sigma–Aldrich, St. Louis, USA (SAB1300907)
5-hydroxytryptamine (5-HT)	Rabbit	Polyclonal IgG	1:10000	Sigma–Aldrich, St. Louis, USA (SAB4501480)

Myelin Basic Protein (MBP)	Rabbit	Polyclonal IgG	1:400	Merck Millipore, Billerica, USA (AB980)
Glial fibrillary acidic protein (GFAP)	Rabbit	Polyclonal IgG	1:1000	Boster, Wuhan, China (PB0046)
Nestin	Rabbit	Monoclonal IgG	1:1000	Sigma–Aldrich, St. Louis, USA (SAB5500150)
S100	Mouse	Polyclonal IgG	1:500	Abcam, London, UK (ab14849)
GAPDH	Mouse	Monoclonal IgG	1:1000 0	Abcam, London, UK (ab8245)
Ionized calcium binding adaptor molecule 1 (IBA-1)	Rabbit	Polyclonal IgG	1:500	Abcam, London, UK (ab178846)
c-fos	Rabbit	Polyclonal IgG	1:800	Abcam, London, UK (ab209274)
Fibronectin (FN)	Rabbit	Polyclonal IgG	1:1000	Abcam, London, UK (ab2413)
Tyrosine hydroxylase (TH)	Rabbit	Polyclonal IgG	1:1000	Abcam, London, UK (ab112)
VGluT1	Mouse	Monoclonal IgG	1:2500	Abcam, London, UK (ab242204)
Laminin (LN)	Rabbit	Polyclonal IgG	1:500	Abcam, London, UK (ab11575)
Growth-related protein 43 (GAP43)	Rabbit	Polyclonal IgG	1:500	Abcam, London, UK (ab16053)
DyLigh 405 Goat Anti-Rabbit secondary antibody	Goat	Polyclonal IgG	1:500	Jackson ImmunoResearch, West Grove, USA (111-475-003)
Alexa 647 conjugated anti rabbit secondary antibody	Goat	Polyclonal IgG	1:500	Jackson ImmunoResearch, West Grove, USA (100699)
Cy3 conjugated anti rabbit secondary antibody	Goat	Polyclonal IgG	1:300	Jackson ImmunoResearch, West Grove, USA (711-165-162)

Cy3 conjugated anti mouse secondary antibody	Goat	Polyclonal IgG	1:300	Jackson ImmunoResearch, West Grove, USA (115-165-146)
DyLigh 405 Goat Anti-Mouse secondary antibody	Goat	Polyclonal IgG	1:200	Jackson ImmunoResearch, West Grove, USA (115-475-146)
Goat Anti-Mouse (HRP)	Goat	Polyclonal IgG	1:5000	Abcam, London, UK (ab6789)
Goat Anti-Rabbit (HRP)	Goat	Polyclonal IgG	1:10000	Abcam, London, UK (ab6721)

Tissue-Engineered Neural Network Graft Relays Excitatory Signal in the Completely Transected Canine Spinal Cord

Bi-Qin Lai#, Ming-Tian Che#, Bo Feng, Yu-Rong Bai, Ge Li, Yuan-Huan Ma, Lai-Jian Wang, Meng-Yao Huang, Ya-Qiong Wang, Bin Jiang, Ying Ding, Xiang Zeng, Yuan-Shan Zeng**

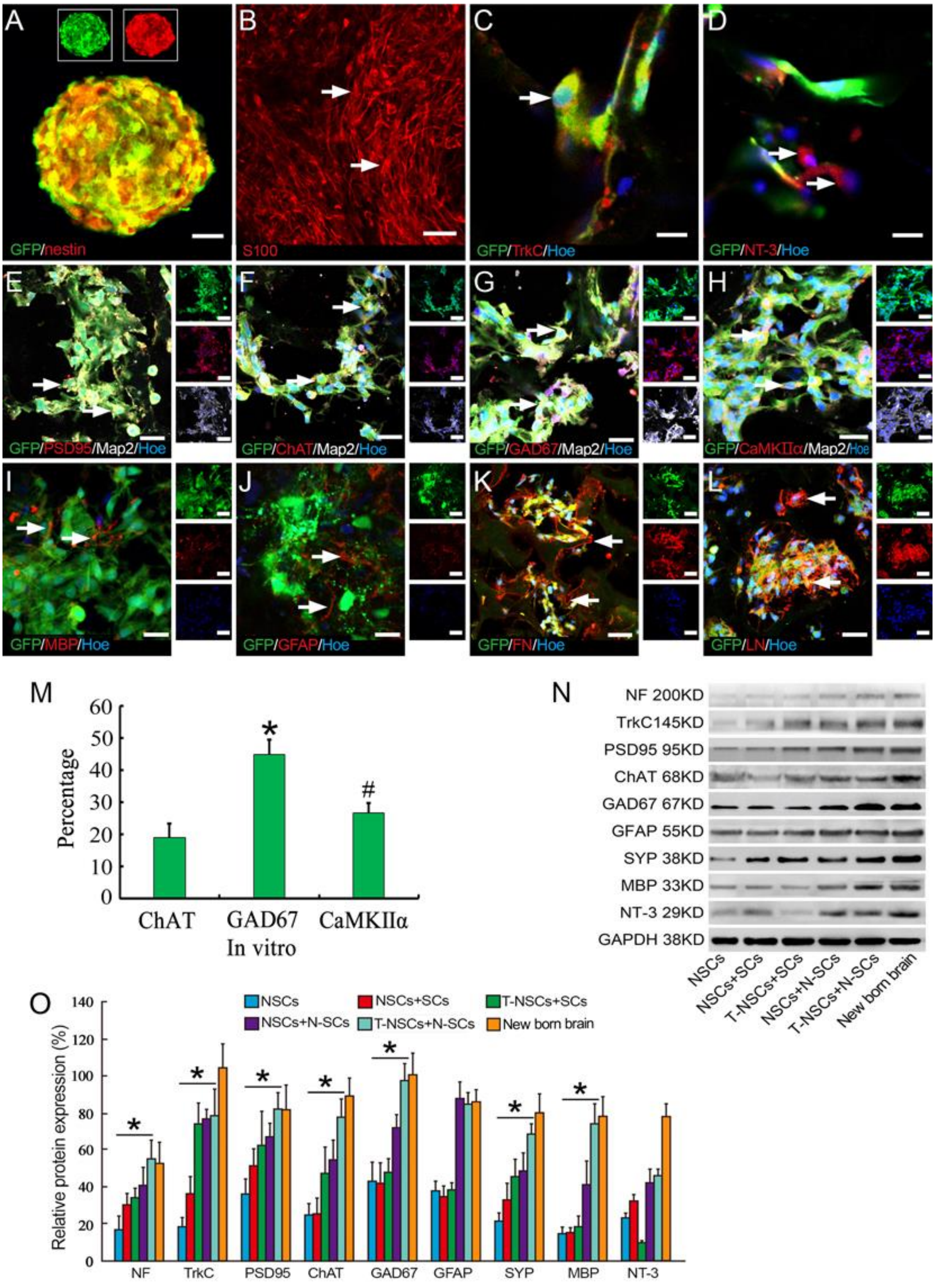

Figure S1. Construction and phenotypic identification of NSC-derived NN tissue *in vitro*. (**A**) A large portion of cells in a neurosphere transfected with pLent-TrkC-GFP expressed the NSC marker nestin. (**B**) SCs expressed S-100 (arrows). TrkC-NSCs (arrow in **C**) and NT-3-SCs (arrow in **D**) were seeded onto 3D CS scaffolds. (**E**) NSC-derived cells (GFP positive, arrows) in the NN tissue were double-immunostained for Map2 and PSD95. GFP- and Map2-positive cells in the NN tissue expressed ChAT (arrows in **F**), GAD67 (arrows in **G**), or CaMK II α (an excitatory neuron marker, arrows in **H**). (**I**) and (**J**) Non-NSC-derived cells (GFP-negative) expressed MBP (arrows in **I**) or GFAP (arrows in **J**). (**K**) and (**L**) Immunofluorescence staining for two major ECM components, fibronectin (FN, arrows in **K**) and laminin (LN, arrows in **L**), in the NSC-derived NN tissue. Hoe = Hochest33342. (**M**) Bar chart showing the percentages of ChAT-, GAD67-, and CaMK II α-positive cells among all GFP-positive cells. * and # symbols indicate $P < 0.05$ ($n = 5$) when ChAT was compared with GAD67 or CaMK II α, respectively. (**N**) Expression of relevant neural and exogenous proteins in all groups after 14 days of culture. (**O**) Relative levels of each protein in all groups. *$P < 0.05$ (n=5), when the T-NSCs+N-SCs group was compared with the NSCs, NSCs+SCs, T-NSCs+SCs, and NSCs+N-SCs groups. Scale bars = 50 μm in (**A**), (**B**), (**K**), and (**L**); 10 μm in (**C**) and (**D**); 20 μm in (**E**)-(**J**).

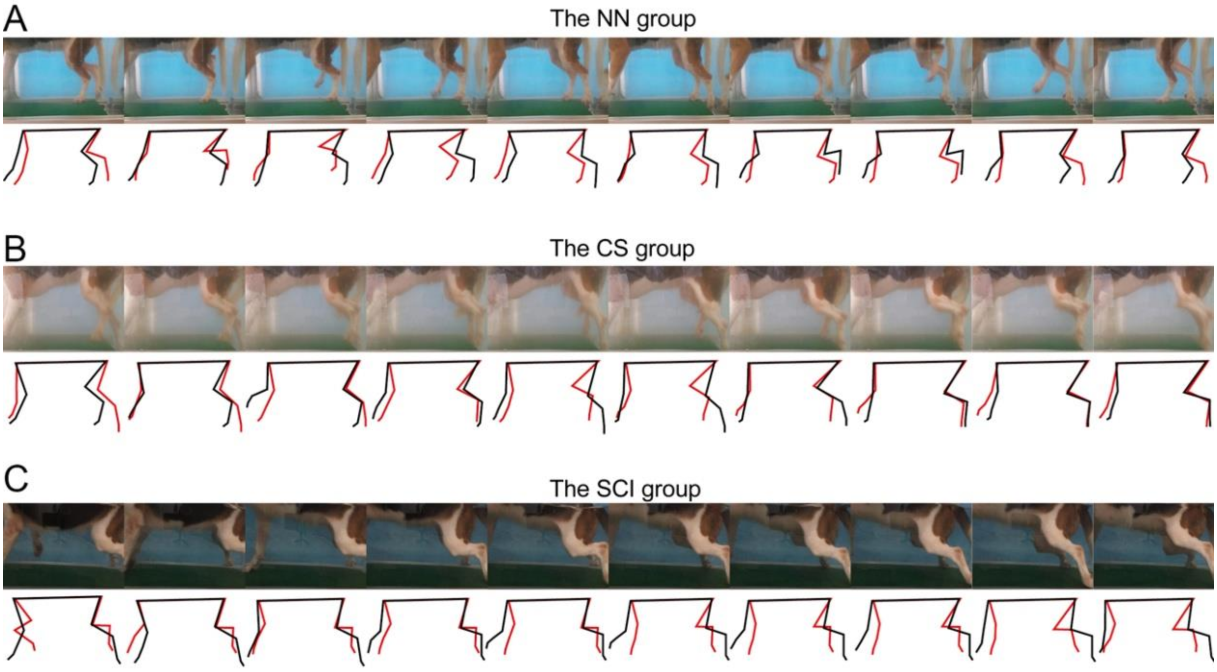

Figure S2. Coordinated locomotion on an underwater treadmill. Canines were placed on a treadmill submerged in warm water 24 weeks after SCI. (**A**) Frequent front-pelvic limb coordinated stepping was observed in the NN group. (**B**) and (**C**) Canines in the CS group (**B**) and SCI group (**C**) did not regain front-pelvic limb coordinated stepping.

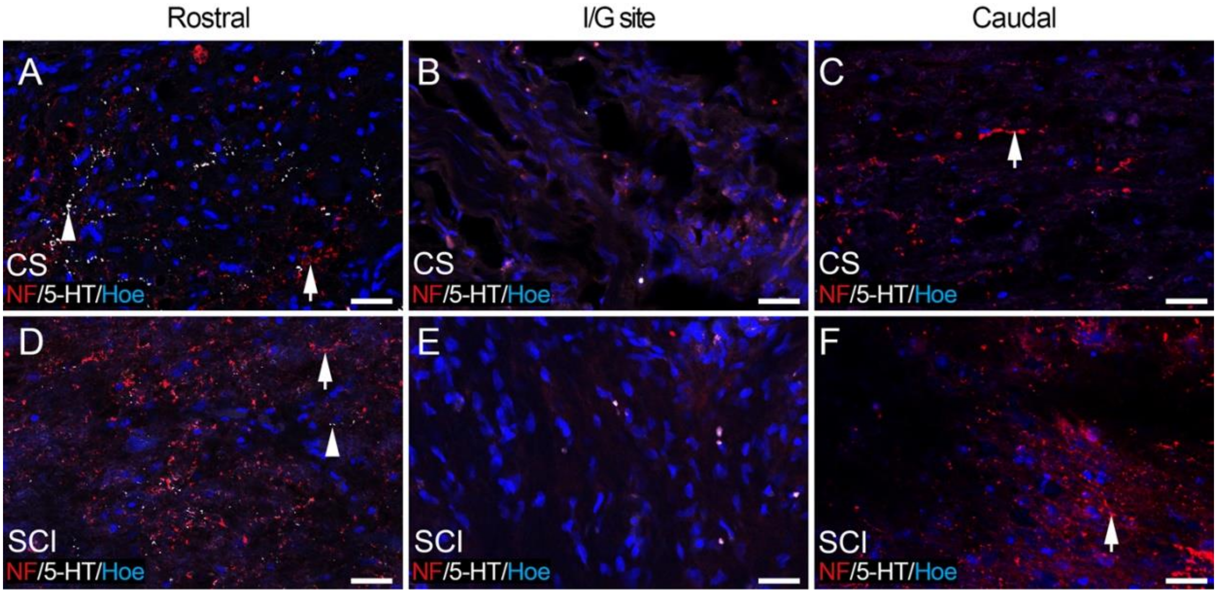

Figure S3. Distribution of NF- and 5-HT-positive nerve fibers in the injured spinal cord 24 weeks following surgery. (**A**) NF- (arrow) and 5-HT- (arrowhead) positive fibers in the rostral site of the CS groups. (**B**) NF- and 5-HT-positive fibers in the I/G site of the CS groups. (**C**) NF- (arrow) and 5-HT-positive fibers in the caudal site of the CS groups. (**D**) NF- (arrow) and 5-HT- (arrowhead) positive fibers in the rostral site of the SCI groups. (**E**) NF- and 5-HT-positive fibers in I/G site of the SCI groups. (**F**) NF- (arrow) and 5-HT-positive fibers in the caudal site of the SCI groups. Hoe = Hochest33342. Scale bars = 40 μm in (**A**)-(**F**).

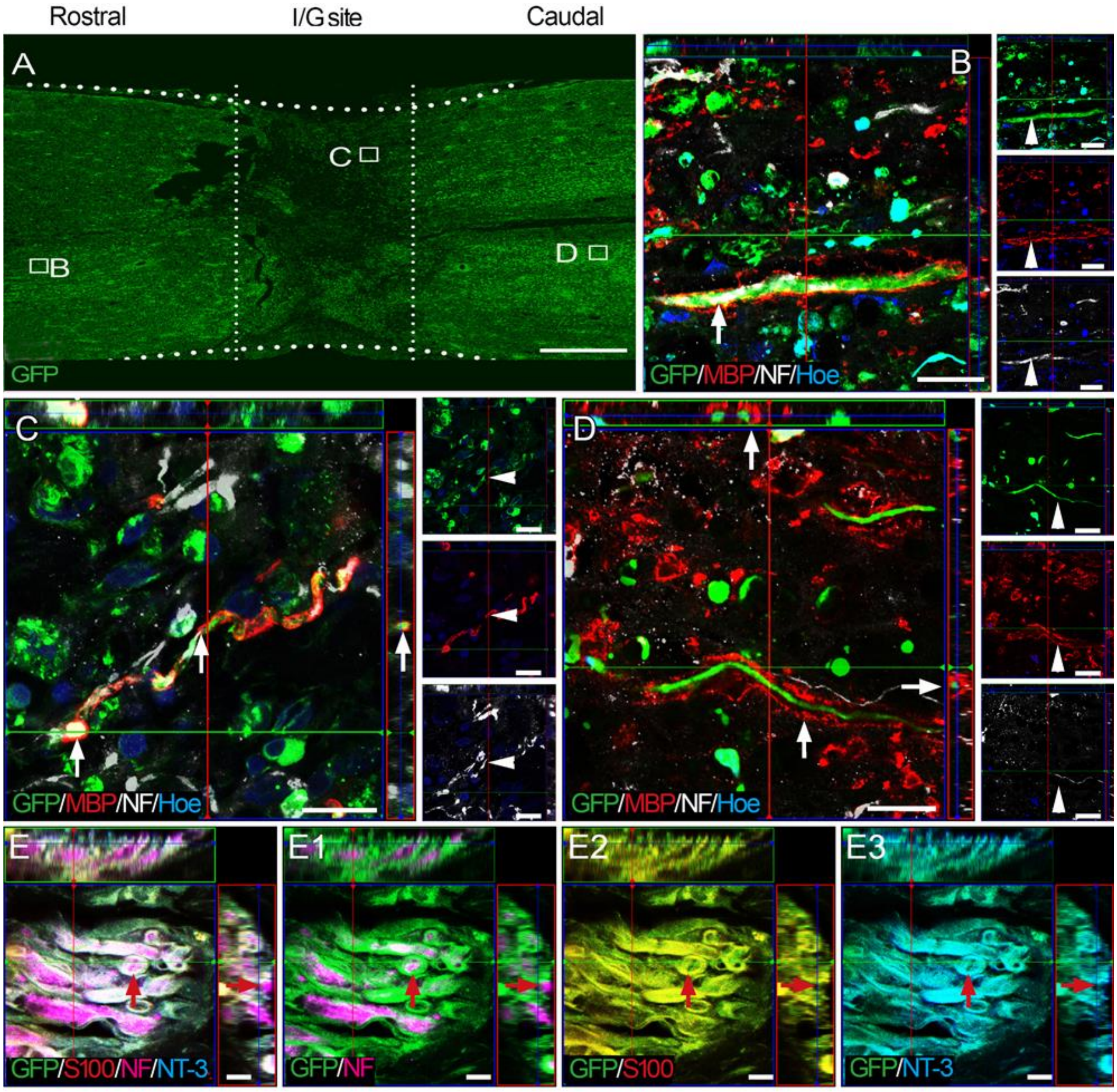

Figure S4. Myelination of the NN tissue transplanted in the injured spinal cord. (**A**) Longitudinal section of an injured spinal cord in the NN group 24 weeks after injury. (**B**)-(**D**) GFP-positive donor neurites were wrapped with MBP-positive myelin sheaths in the adjacent area rostral to the I/G site (NF positive, arrow in **B**), in the I/G site (NF positive, arrows in **C**), and in the adjacent area caudal to the I/G site (arrows in **D**). (**E**) Transplantation of GFP-positive SCs in the NN tissue resulted in formation of SC-derived myelin sheaths that wrapped NF-positive nerve fibers in the I/G site 24 weeks after transplantation. GFP-positive SCs (**E**1) expressed S-100 (**E**2) and NT-3 (**E**3). Hoe = Hochest33342. Scale bars = 2 mm in (**A**); 20 μm in (**B**) and (**D**); 40 μm in (**C**); 5 μm in (**E**)-(**E**3).

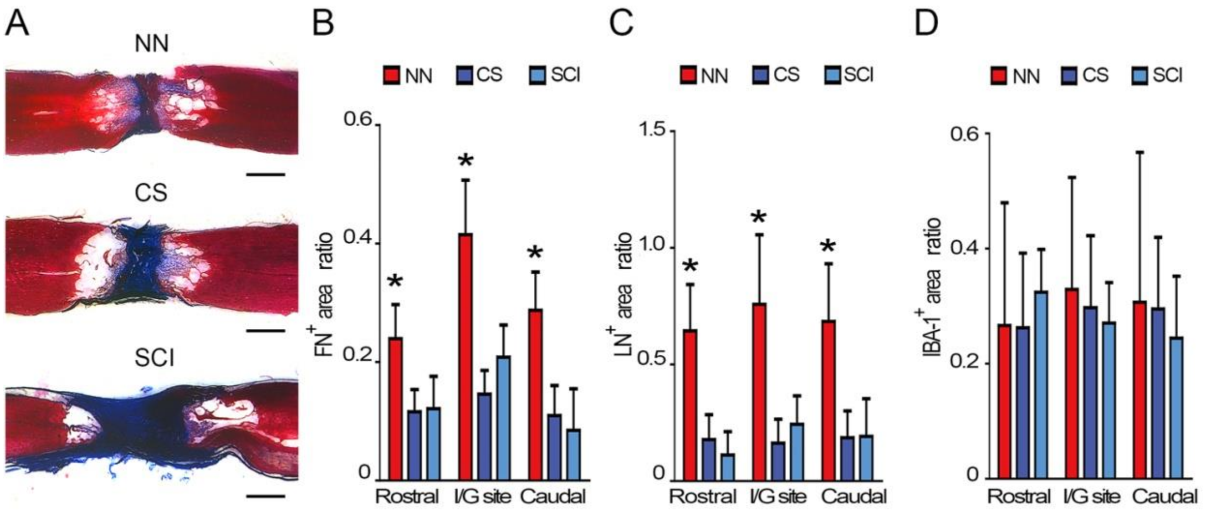

Figure S5. Deposition of ECM and the inflammation 24 weeks after SCI. (**A**) Masson's trichrome staining showed obvious collagen deposition in the I/G site of the spinal cord in the SCI and CS groups, relative to the NN group. (**B**) and (**C**) Histograms showing that FN and LN were extensively expressed at the I/G site of the spinal cord in the NN group, relative to that in the CS or SCI group (*$P < 0.05$). (**D**) There was no statistical difference in IBA-1-positive areas in the areas rostral or caudal to the I/G site, or in the I/G site among the NN, CS, and SCI groups. Scale bars = 1 mm in (**A**).

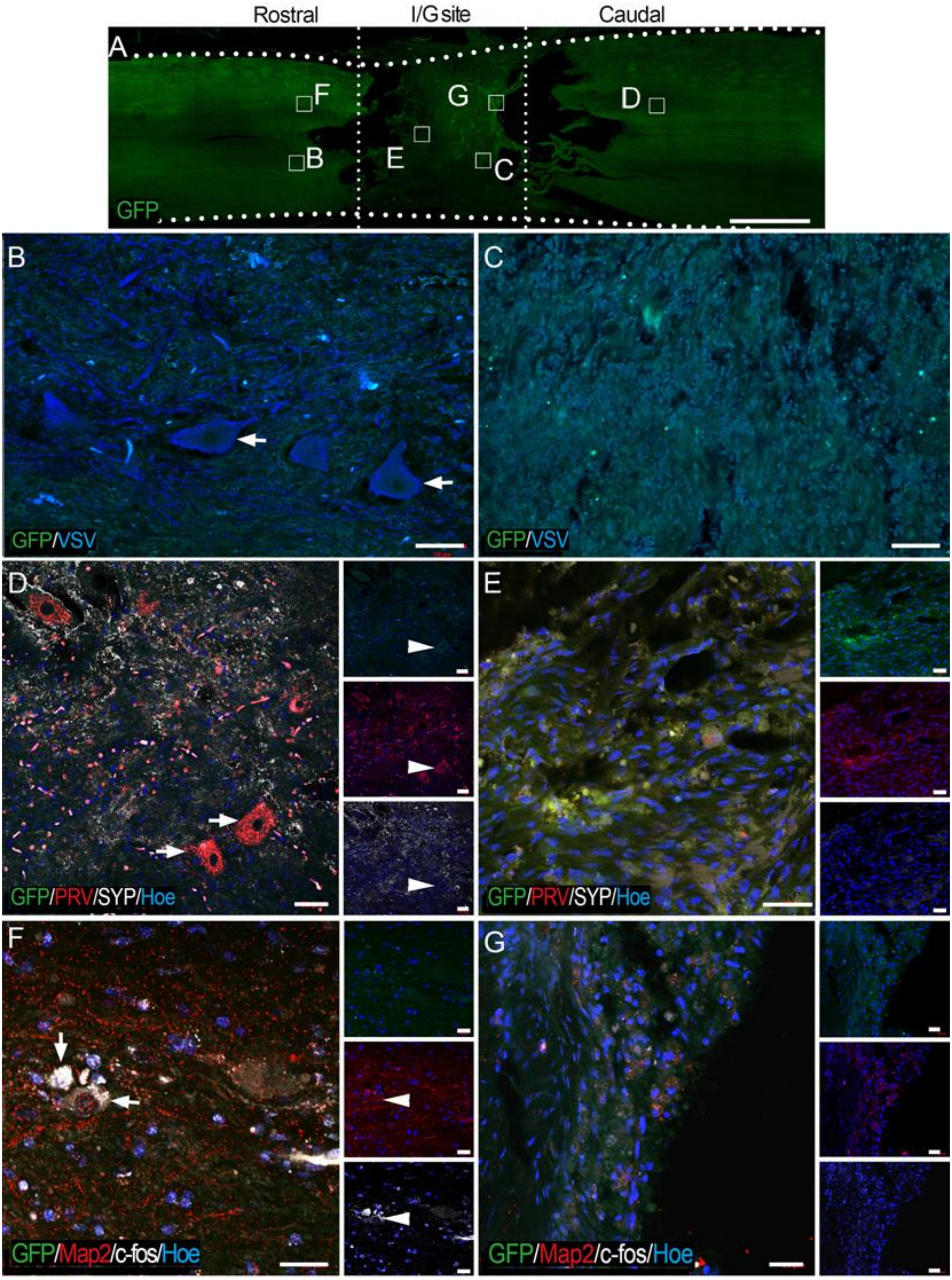

Figure S6. Expression of c-fos, and VSV anterograde and PRV retrograde tracing in the CS group. (**A**) Overview of the longitudinal section of the injured spinal cord. (**B**) and (**C**) Fluorescence of blue fluorescence protein (BFP) encoded by VSV was observed in some

neurons rostral to the I/G site (arrows in **B**), but not in the I/G site or area caudal to the I/G site (**C**). (**D**) and (**E**) Fluorescence of red fluorescence protein (RFP) encoded by PRV was observed in neurons caudal to the I/G site (arrows in **D**), but not in the I/G site or rostral to the I/G site (**E**). (**F**) and (**G**) c-fos immunoreactivity was observed in neurons rostral to the I/G site (arrows in **F**), but not in the I/G site or caudal to the I/G site (**G**). Hoe = Hochest33342. Scale bars = 2 mm in (**A**); 40 μm in (**B**)-(**G**).

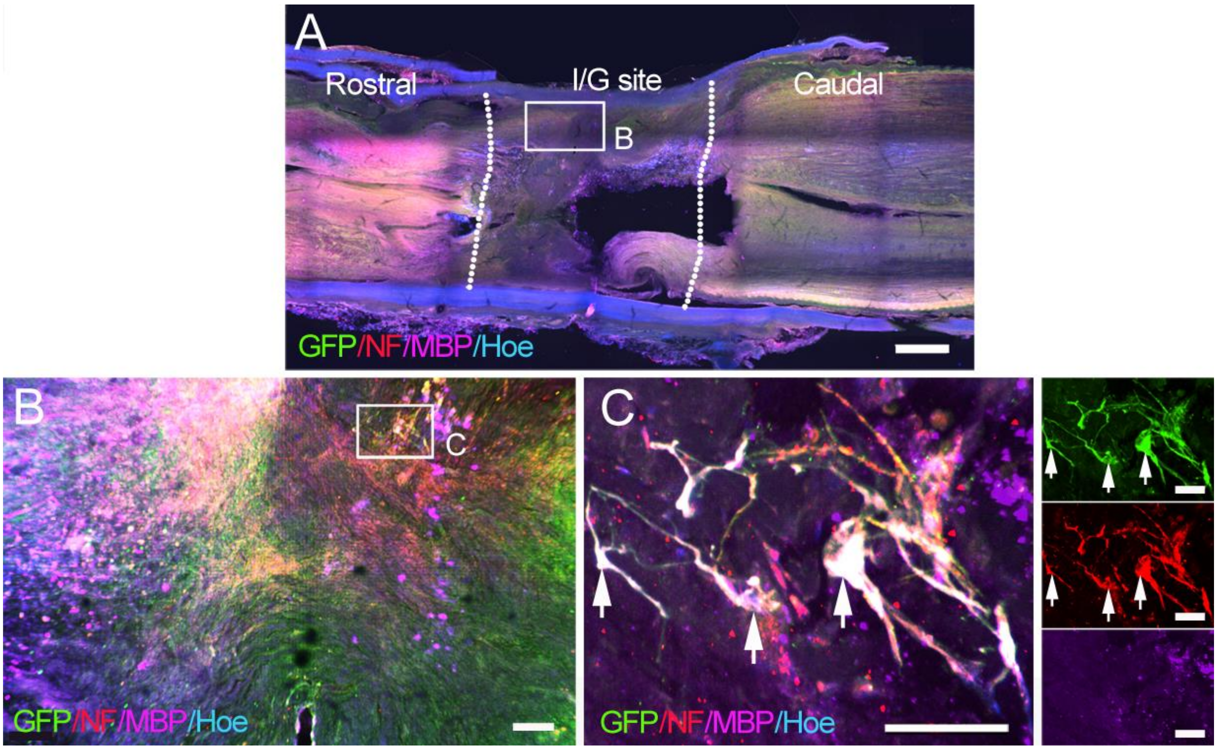

Figure S7. Survival of NN tissue transplanted in the injured spinal cord at 72 weeks. A) Overview of a longitudinal section of the spinal cord segment containing the I/G site in the NN group. B) A portion of GFP-positive donor cells survived in the I/G site. C) A subset of the donor cells maintained phenotypes of neurons (arrows, NF immunopositive). MBP is a marker of myelin sheath. Hoe = Hochest33342. Scale bars = 1 mm in (**A**), 100 μm in (**B**), 30 μm in (**C**).
